# Detection of a Cryptic 25 bp Deletion and a 269 Kb Microduplication by Nanopore Sequencing in a Seemingly Balanced Translocation Involving the LMLN and LOC105378102 Genes

**DOI:** 10.3389/fgene.2022.883398

**Published:** 2022-08-26

**Authors:** Yanan Wang, Zhenhua Zhao, Xinyu Fu, Shufang Li, Qiuyan Zhang, Xiangdong Kong

**Affiliations:** Genetic and Prenatal Diagnosis Center, Department of Obstetrics and Gynecology, the First Affiliated Hospital of Zhengzhou University, Zhengzhou, China

**Keywords:** balanced translocation, nanopore sequencing, cryptic duplication, deletion, karyotype analysis, CNV

## Abstract

Preimplantation genetic testing plays a critical role in enabling a balanced translocation carrier to obtain the normal embryo. Identifying the precise breakpoints for the carriers with phenotypic abnormity, allows us to reveal disrupted genes. In this study, a seemingly balanced translocation 46, XX, t (3; 6) (q29; q26) was first detected using conventional karyotype analysis. To locate the precise breakpoints, whole genomes of DNA were sequenced based on the nanopore GridION platform, and bioinformatic analyses were further confirmed by polymerase-chain-reaction (PCR) and copy number variation (CNV). Nanopore sequencing results were consistent with the karyotype analysis. Meanwhile, two breakpoints were successfully validated using polymerase-chain-reaction and Sanger Sequencing. LOC105378102 and LMLN genes were disrupted at the breakpoint junctions. Notably, observations found that seemingly balanced translocation was unbalanced due to a cryptic 269 kilobases (Kb) microduplication and a 25 bp deletion at the breakpoints of chromosome (chr) 6 and chr 3, respectively. Furthermore, 269 Kb microduplication was also confirmed by copy number variation analyses. In summary, nanopore sequencing was a rapid and direct method for identifying the precise breakpoints of a balanced translocation despite low coverage (3.8×). In addition, cryptic deletion and duplication were able to be detected at the single-nucleotide level.

## Introduction

Balanced translocation is a kind of common chromosomal rearrangement. After two chromosomes are broken, they exchange genetic materials with each other to form two new derived chromosomes. Most balanced translocations have no serious influences on gene expression and development because the total number of genes does not change. The incidence of balanced translocation in the population is 0.2%, but for patients with a history of recurrent miscarriages, the incidence is increased to 2.2% (Mackie Ogilvie and Scriven, 2002). Generally, carriers have a normal phenotype, but they are at high risk of facing reproductive problems such as infertility, miscarriage, and unbalanced gametes. In the meiosis process, two translocated chromosomes will form quadrivalent, which may produce 18 gamete types depending on the way they are separated. Only one type is perfectly normal, and one is a balanced translocation carrier.

The most common method to detect the balanced translocation is known as the G-banded karyotype analysis, this method can detect the chromosomal structural variation with a resolution of 5–10 megabases (Mb) (Talkowski et al., 2012). Karyotype analysis is the most cost-effective means for balanced translocation, and still cannot be displaced by other technologies (Wilch and Morton, 2018). It is also unable to readily distinguish between similar banding patterns and sizes (Dong et al., 2018a; Dong et al., 2018b). Fluorescence *in situ* hybridization (FISH) is another widely used technology that targets specific genes or locations with a resolution of <1 Mb. The limitation is that probe design is complex and time-consuming (Beyazyurek et al., 2010; Fiorentino et al., 2010). With the development of sequencing technology, low coverage mate-pair whole genome sequencing (WGS) as a complement to conventional karyotype analysis is used for defining precise breakpoints. Talkowski et al. discovered that a breakpoint directly disrupted the CHD7 gene, a causal locus in the CHARGE syndrome (Talkowski et al., 2012). There have been many similar studies in the past decade. Following microdissecting junction region and next-generation sequencing (Jancuskova et al., 2013), Hu et al. combined single-nucleotide polymorphism (SNP) linkage analyses with long-range PCR to identify the carrier and normal embryos, and prenatal diagnosis for fetuses confirmed the feasibility of this strategy (Hu et al., 2016). With the successful application of SNP microarray, several teams have detected embryo biopsy samples for the translocation carrier, this is an indirect and efficient method to distinguish the normal and carrier embryos based on haplotype phasing. However, a translocation carrier’s family member or unbalanced embryos are required as a reference to obtain the SNP genotype (Idowu et al., 2015; Treff et al., 2016; Yao et al., 2016; Xu et al., 2017; Zhang et al., 2017; Li et al., 2021). Moreover, the identification of precise breakpoints often requires multiple methods due to technique restrictions. Recently, Wang et al. used a single-molecule optimal mapping technology with the Bionano platform to identify nine balanced reciprocal translocations. Breakpoint regions were finely mapped to small regions of approximately 10 Kb (Wang et al., 2020). Similarly, another study detected a Marfan syndrome caused by the disruption of FBN1 gene due to a reciprocal chromosome translocation with the help of optical genome mapping (Schnause et al., 2021). In recent years, third-generation sequencing technology has developed rapidly due to its long-read length. For structural variations including the balanced translocation, several studies have successfully detected chromosomal rearrangement using Oxford Nanopore or the PacBio SMRT platform (Cretu Stancu et al., 2017; Sedlazeck et al., 2018; Dutta et al., 2019; Hu et al., 2019; Chow et al., 2020; Liu et al., 2021; Cheng et al., 2021; Pei et al., 2021; Wang et al., 2021).

In this study, we aim to locate the precise breakpoints of a balanced translocation carrier with a Nanopore GridION device according to the results of karyotype analysis, provide effective genetic counseling for the patient, help them distinguish between normal and carrier embryos in the following assisted reproduction, and block the transmission of chromosomal translocation to the next generation (Madjunkova et al., 2020).

## Materials and Methods

### Patient Information and Ethics Statement

The use of patient blood was reviewed and approved by the Research and Clinical Trials Ethics Committee of the First Affiliated Hospital of Zhengzhou University, and consent was obtained by the index case. The case patient had two adverse pregnancies. They said that they had conceived a child with a 6q26qter 6.5 MB heterozygous deletion, so the pregnancy had been terminated. Before the assisted reproduction, the ultrasound examination indicated that there were no obvious abnormalities in cardiac structure and function. The electrocardiograph was also normal. Digital radiography (DR) examination showed no obvious abnormality of the cardiopulmonary diaphragm. After hysteroscopic tubal catheterization, both fallopian tubes were unobstructed and the uterine cavity was normal. They are now continuing to seek assisted reproduction for a healthy pregnancy in our hospital.

### Karyotype Analysis

We collected 5 ml of venous blood and incubated the leukocytes at 37°C for 66–72 h. As per the usual procedure, gentle agitation of the culture flask every 24 h promoted cell growth. We added 10 μL of colchicine and culture for an additional 1 h. The cells were then treated with 10 ml of hypotonic KCl and we then fixed the cells twice with freshly prepared fixative. The cells were stained with Giemsa following trypsin treatment. Photograph and identification of specific chromosomes was undertaken using the automatic chromosomal analysis system from Leica.

### DNA Extraction

Genomic DNA was extracted from 200 μL of frozen blood. According to the instructions of the Qiaamp blood DNA mini kit, 200 μL of high-quality DNA was obtained. The concentration of DNA was quantified using a Qubit 2.0 fluorometer with a dsDNA HS assay kit (ThermoFisher Scientific). DNA purity and length were measured with Nanodrop 2000 (ThermoFisher Scientific) and the Qseq100 analysis system, respectively.

### Library Preparation and Nanopore Sequencing

Based on the pilot experiment, 1.1 μg of gDNA were prepared for library construction according to the manufacturer’s instructions using a ligation sequencing kit SQK-LSK109 (Oxford Nanopore Technologies). DNA was cleaned up with Agencourt AMPure XP magnetic beads (Beckman Coulter) following end repair and dA-tailing. Then, 1 μL eluted DNA was quantified to calculate the yield. After adapter ligation, all DNA fragments were enriched equally. Generally, 25% of DNA was lost at this step. Different from the standard protocol, we loaded 100 fmol of the final library onto the R9.4.1 flow cell of the nanopore GridION X5 platform.

After running for about 60h, the raw electrical signal (.fast5) was converted to base sequences (.fastq) through superhigh-accuracy basecalling mode. Reads with mean_qscore_template of more than 8 were defined as a “pass”. The clean data were aligned to the human reference genome (GRCh38/hg38) using Minimap2 (Li, 2018). The filter parameter mapQ was set up more than 40. Variant calling was performed with Sniffles (Sedlazeck et al., 2018), and the bioinformatics workflow was based on a custom Python script, as shown in the Supplementary Materials of this article. Reads aligned to chr 3 and chr 6 simultaneously were extracted and analyzed manually using the NCBI blast tool (https://blast.ncbi.nlm.nih.gov/Blast.cgi). Finally, reads were visualized by uploading a. Sam or. bam file to the online tool genome Ribbon (Nattestad et al., 2021) (https://genomeribbon.com/).

### PCR and Sanger Sequencing

The breakpoints were further confirmed by PCR, and Primer Premier 6 was used for designing primers. For the normal chr 3 and chr 6, 2 Kb bases flanking the potential breakpoints were directly retrieved from NCBI. For the derived chr 3 and chr 6, 2 Kb bases flanking the breakpoints were manually spliced. The primes used in this study were as follows. F1: AAT​TCA​AAA​CTC​TTG​TGT​CAC​TTT​G, R1: ACA​TGT​TAC​CAC​AAT​TCA​TGC​ACT; F2: GGA​GAG​ACA​AAG​GGA​AGT​GTC​A, R2: CCC​CTT​TAC​AAT​CCC​AAA​TTC​AGG; F3: AGT​AGT​CTG​TAG​AGT​CTG​CTA, R3: GTC​TTG​TCT​CAC​GCT​CAG; F4: GCA​GCT​TTG​AAA​ATG​GTT​ACT​TGG, R4: ACA​ATT​TGA​GAG​AAA​GTT​GCA​GGA. A prepared mix on ice was: gDNA 50 ng, forward primer 1 μL, reverse primer 1 μL, 2×Taq master mix (Tiangen) 10 μL, and add ddH_2_O to 20 μL. The cycling conditions were set as follows: 5 min at 95°C, then 32 cycles of 94°C for 30s, 57°C for 30s and 72°C for 30 s, and finally, 5 min at 72°C. Amplicons were visualized on 1% agarose gel and further confirmed by Sanger Sequencing (Sangon Biotech).

### CNV Analyses

CNV-seq was performed according to the instructions of the CNV library sequencing kit (Berry Genomics, Beijing). For the CNV, genomic DNA (gDNA) needed to be purified before library preparation. We used the column purification method and the purification efficiency was 60%. gDNA was fragmented by enzyme digestion, followed by the addition of dA-tailing and ligation of barcodes and sequencing adapters. The concentration of each sample was determined by quantitative real-time PCR (qPCR), and different volumes of barcoded gDNA were mixed according to the sample concentration. The sequencing platform was Illumina NextSeq CN500, and the sequencing type was SE45 (single-ended sequencing, read length 45bp). The average sequencing depth was 0.1×. Firstly, the raw sequencing reads were aligned to human genome reference sequence version GRCh37/hg19. Meanwhile, CNVs were obtained based on the following algorithm.

$\mathrm{copy}N_{\mathrm{bin}}=\frac{\mathrm{obsR}C_{\mathrm{bin}}}{\mathrm{refR}C_{\mathrm{bin}}}\times \mathrm{copy}N_{\mathrm{chrom}}\cdot \mathrm{copy}N_{\mathrm{bin}},\mathrm{obsR}C_{\mathrm{bin}},\mathrm{refR}C_{\mathrm{bin}}\;\mathrm{and}\;\mathrm{copy}N_{\mathrm{chrom}}$ represents the copy number of bin, the reads number of detected sample and control, and the theoretical copy number of the chromosome at which bin is located, respectively. The genome coordinates between reference versions GRCh37/hg19 and GRCh38/hg38 were converted with the tool Lift Genome Annotations (https://genome.ucsc.edu/cgi-bin/hgLiftOver). The resolution of CNV was 100 Kb.

### Software and Database

Figures were generated with GraphPad Prism 9. In this study, the NCBI blast tool was used for manual alignment. Reads were visualized using the online tool Ribbon. Repetitive sequences were analyzed using RepeatMasker tool (http://repeatmasker.org). Sanger sequencing was visualized using Snapgene, and primers were designed with Primer Premier 6.

## Results

### Breakpoint Determination by Karyotype Analysis and Nanopore Sequencing

**FIGURE 1:**
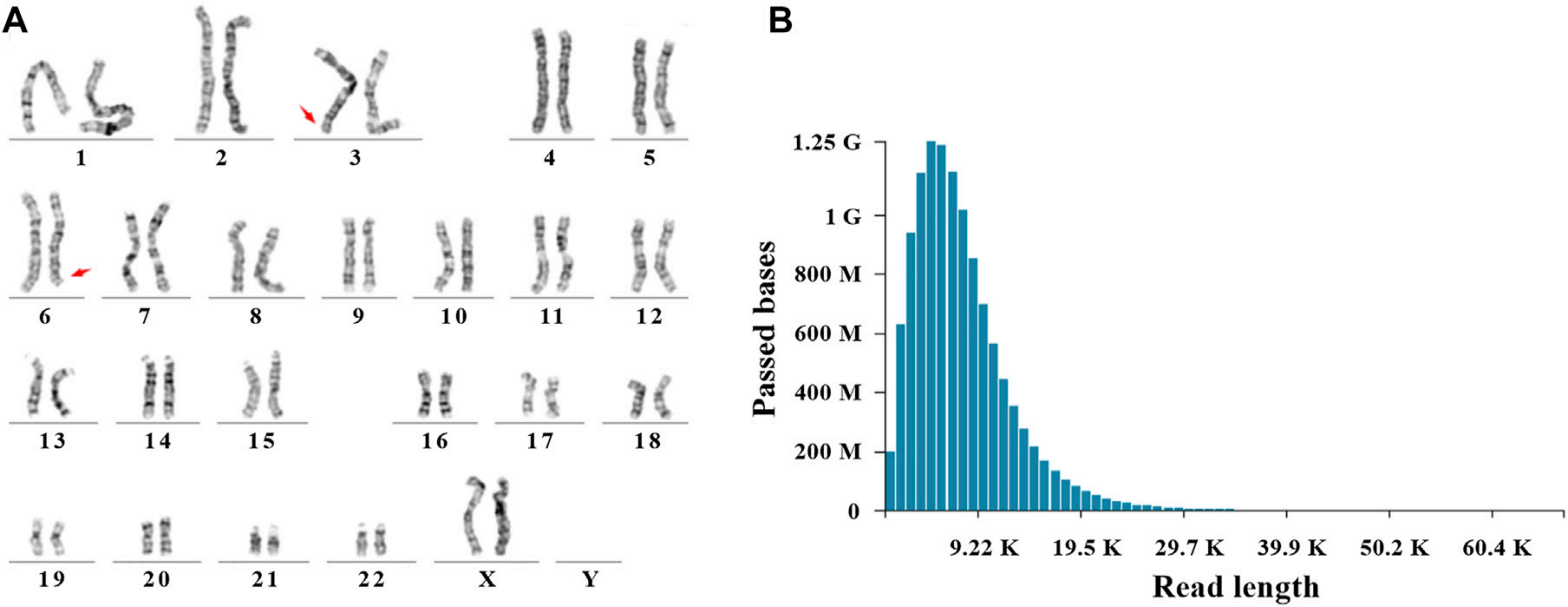
Cytogenetic analysis and Nanopore sequencing. **(A)** A balanced translocation between chr 3 and chr 6 was shown by G-banded karyotype. Red arrows indicated the breakpoints (q29 of chr 3 and q26 of chr 6). **(B)** Read length and passed bases were displayed.

**FIGURE 2:**
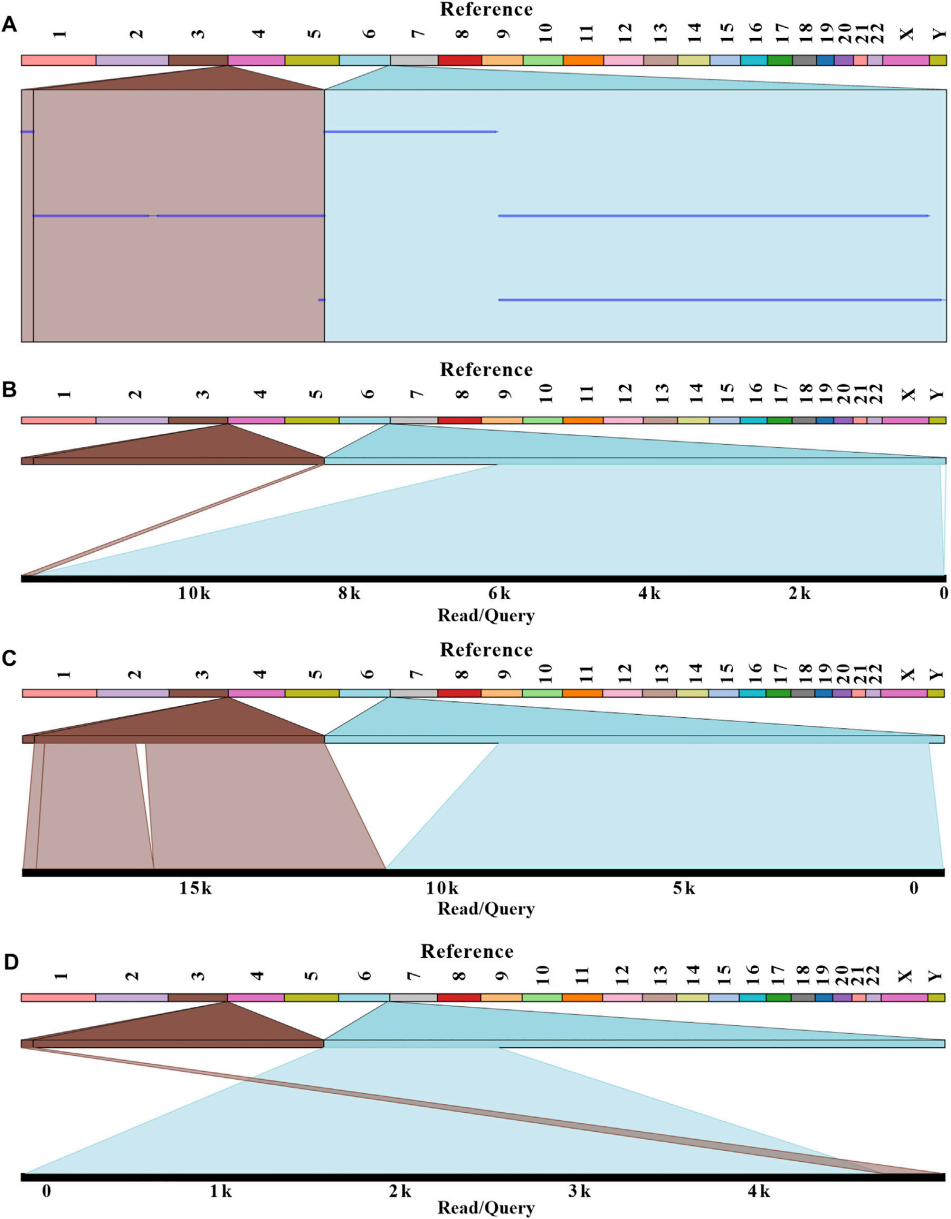
Balanced translocation was visualized with Ribbon. Three reads related to the breakpoints were aligned with a human reference genome (GRCh38/hg38). **(A)** The summary of three reads mapping. **(B–D)** The detailed alignment results for each read were shown. The breakpoints were located at positions: chr 3:198,009,533; chr 6:164,036,632 for B and C; chr 3:197,740,493; and chr 6:164,036,607 for D.

After genetic counseling, conventional cytogenetic analysis was performed for this patient. G-banded results revealed a balanced translocation between chr 3 and chr 6, and the karyotype was 46, XX, t (3; 6) (q29; q26), as shown in Figure 1. In order to identify the breakpoints of chr 3 and chr 6, the gDNA library was constructed and sequenced on the nanopore GridION platform. After running for about 60h, we obtained 11.8 gigabases (Gb) of passed bases and the average sequencing coverage was 3.8×. The estimated N50 was 6.57 Kb (Supplementary Figure S1). The sequencing coverage was relatively low and unfriendly to bioinformatic analysis. Based on the limited data, a custom Python script was developed for the downstream analysis. The bioinformatic pipeline included two alignment programs (Minimap2 and ngmlr) and a variant calling tool Sniffle. As much data as possible were extracted through two combinations. Considering the resolution of karyotype analysis, all translocations (15 Mb) flanking the q29 of chr 3 and q26 of chr 6 were analyzed. Finally, eight potential breakpoints were discovered. Among them, the physical locations of two breakpoints (chr3:198,009,533 and chr6:164,036,632) were in accordance with karyotype analysis (Figure 2A). Two reads supported the above breakpoints. Unexpectedly, another read was located at the breakpoints of chr3:197,740,493 and chr6:164,036,607 (Figures 2B–D). The nucleotide sequences of the abovementioned three reads are provided in the Supplementary Materials. The remaining six breakpoints were located near the target position, and detailed physical locations were shown in Supplementary Table S1.

### PCR and Sanger Sequencing

**FIGURE 3:**
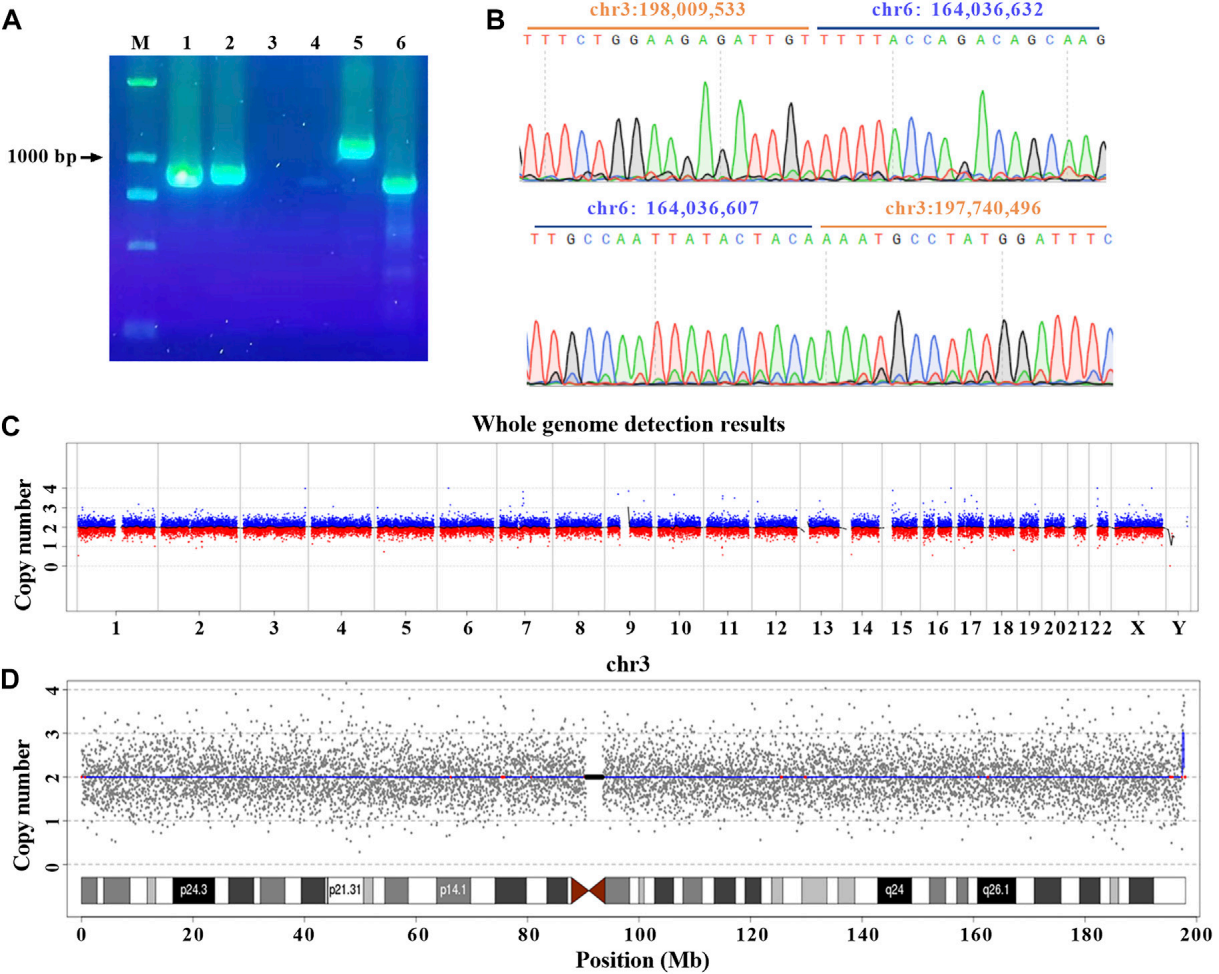
Cryptic deletion and microduplication were confirmed with PCR and CNV. **(A)** gDNA were extracted from 200 μL peripheral blood. PCR was performed with specific primers designed for normal and rearranged chr 3 and chr 6. Amplicons (lanes 1 and 2) were obtained for normal chr 3 (primer pair F1/R1) and chr 6 (primer pair F2/R2) of the patient. Breakpoints of chr 3 (lanes 3 and 5 with primer pair F3/R3) and chr 6 (lanes 4 and 6 with primer pair F4/R4) were amplified for healthy control and the patient, respectively. All primer sequences were shown in materials and methods. M: DNA marker. **(B)** Two breakpoints were validated by Sanger Sequencing. **(C)** Overview of the low-coverage WGS. **(D)** Low-coverage WGS for chr 3. The blue line indicated the abnormal copy number.

To further confirm the precise breakpoints of balanced translocation, PCR and Sanger Sequencing were conducted for each site with specific primers (Supplementary Figure S2 and Supplementary Table S2). The results indicated no expected PCR bands were generated for six potential breakpoints, so we believe the corresponding translocations were false-positive (Supplementary Figure S3). Meanwhile, we got the specific amplicons at the breakpoints of chr3:198,009,533 and chr6:164,036,607 for this translocation carrier. 1,152 bp and 872 bp of PCR products were shown in lanes 5 and 6, respectively. Taking gDNA of a healthy control as a PCR template, no amplicons were generated around the breakpoints of chr 3 and chr 6 (lanes 3 and 4). For the normal chr 3 and chr 6 of this carrier (lanes 1 and 2), 891 bp and 906 bp of PCR products were also obtained (Figure 3A). PCR amplicons were further confirmed by Sanger Sequencing (Figure 3B).

### Deletion and 269 Kb Microduplication occurred at the Breakpoints

Based on the results of nanopore sequencing, we found that the seeming balanced translocation identified by karyotype analysis was actually unbalanced. The q26 of chr 6 was broken and linked to q29 of chr 3. During the process, a 25 bp fragment was deleted (Figure 3B). For chr 3, a breakage was at the physical location of 198,009,533, then a 269 Kb microduplication occurred and was translocated to the q26 of chr 6. Different from the karyotype analysis, nanopore sequencing revealed a cryptic deletion and a large microduplication at the single-nucleotide resolution. To further validate the presence of 269 Kb microduplication and the accuracy of bioinformatic analysis, CNV was performed with a resolution of 100 Kb. As listed in Supplementary Table S2, eight deletions or duplications were detected throughout the whole genome (Figure 3C and Supplementary Table S3). We mainly focused on the variations of chr 3 and chr 6. In accordance with nanopore sequencing, a 280 Kb microduplication was identified at the q29 region of chr 3 (Figure 3D).

### Gene Structure Around the Breakpoints

**FIGURE 4:**
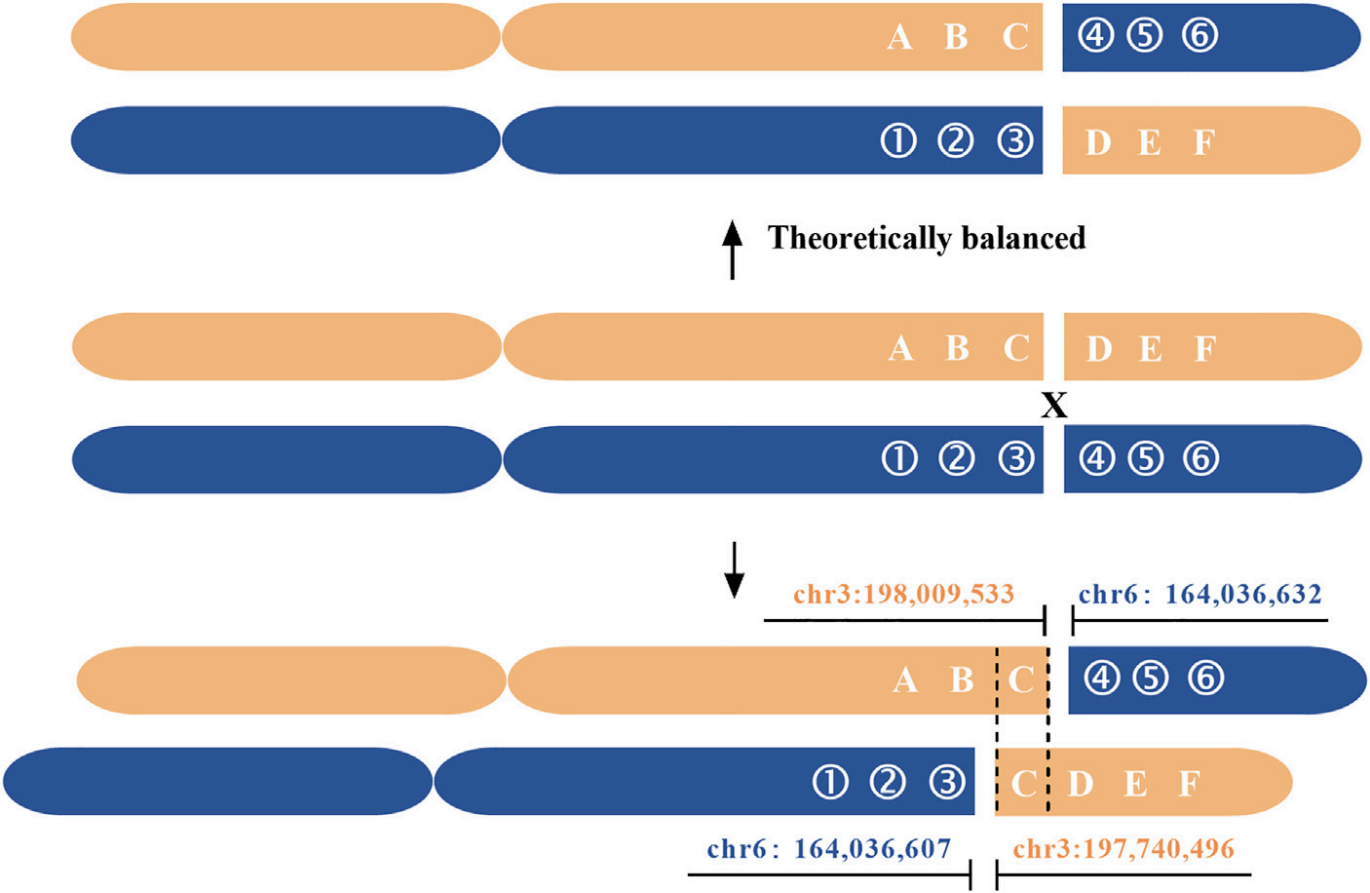
Breakpoint delineation of a seemingly balanced translocation based on nanopore sequencing. Gray and blue represent chr 3 and chr 6, respectively. The arrows point to the breakpoints, and the region between solid lines indicates the 269 Kb microduplication. The dashed line indicates the 25 bp deletion. Nanopore sequencing revealed that the seemingly balanced translocation was unbalanced.

The genomic regions (5 Kb) flanking the breakpoints were analyzed using an online tool RepeatMasker. We revealed that the breakpoint of chr 3 was located at a long interspersed nuclear element (LINE), furthermore, there were a large number of LINEs and short interspersed nuclear elements (SINEs) around the breakpoint. The highly repetitive elements were also a challenge for PCR confirmation. Different from chr 3, the breakpoint of chr 6 was not located at repetitive regions, and it disrupted the LOC105378102 gene, which encoded long non-coding RNA (lncRNA). The breakpoint of chr 3 disrupted LMLN gene, also known as leishmanolysin like peptidase (Supplementary Figure S4). The disruption or fusion of two genes didn’t lead to phenotypic abnormity. Additionally, a microduplication from chr3:197,740,496 to 198,009,533 contained five true genes and two pseudogenes (Figure 4). Because the patient in this study had a normal phenotype, the effects of chromosomal rearrangement on gene function were not further discussed.

## Discussion

As mentioned above, we successfully obtained 11.8 Gb with a sequencing coverage of 3.8×, which was similar to a study by Chow et al. (2020). Compared to other studies (Hu et al., 2019; Cheng et al., 2021), sequencing coverage was very low, and there was a high risk that breakpoints were not captured by limited reads. After starting the sequencing, we found the number of active nanopores declined sharply, and the duty time was shorter than expected. The actives nanopores seemed to be destroyed by something. We speculated the reason may be impure gDNA. The rapid loss of active pores was the main reason for low sequencing depth. To improve the sequencing coverage, a second sequencing running may be used for one sample with another flow cell. Inevitably, this strategy will increase the cost of sequencing. At present, the relatively low coverage made it challenging for widespread clinical applications of third-generation sequencing because of high-cost consideration. Even so, the throughput in this study was enough for us to finish the downstream bioinformatic analysis. Three reads spanning the breakpoints of chr 3 and chr 6 were extracted. PCR and Sanger sequencing also confirmed the presence of two breakpoints.

In comparison, our bioinformatic analyses were based on chromosomal karyotype. Considering the human error of karyotype analyst, all reads flanking the target position were extracted. This method ensured the accuracy of bioinformatics and increased the workload of subsequent validation. In this study, eight potential breakpoints were identified with nanopore sequencing, but out of eight breakpoints, six cannot be supported by PCR. Therefore, these sites were defined as false-positive. We need to optimize the analysis workflow and improve the sequencing coverage to filter false-positive reads in the next work.

Overall balanced translocation was generally involved in two chromosomes, but some complex chromosomal rearrangements may generate multiple breakpoints (Wang et al., 2014; Li et al., 2020; Tan et al., 2020). For this condition, breakpoint analyses were more complex with other approaches, such as karyotype, FISH, and SNP array. Sequencing was capable of providing a more accurate description of balanced translocation and revising the karyotype results at the single-nucleotide resolution (Talkowski et al., 2012; Dong et al., 2019). In addition to identifying the breakpoints, nanopore sequencing was also able to detect cryptic deletion and duplication directly. Studies have shown that some apparently balanced translocations were prone to gene deletion or disruption (De Gregori et al., 2007), and duplication at the breakpoint junction also occurred but was not common (Howarth et al., 2011). In our study, a 25 bp deletion and a 269 Kb microduplication were at the breakpoint junction. Luckily, they did not have an obvious effect on the patient’s health. For the individuals with phenotypic abnormity, the incidence of which was 6.1% in the population with apparently balanced rearrangement (Wapner et al., 2012). Chromosomal rearrangement disrupted the causal gene, and precise breakpoints were especially critical for disease diagnosis (Redin et al., 2017; Aagaard et al., 2020; Bonaglia et al., 2020; Guenzel et al., 2021). As shown in our study, breakage often occurred at highly repetitive regions, and this highlighted another advantage of nanopore sequencing relative to short-read sequencing (Aristidou et al., 2017). Taken together, third-generation sequencing was well suitable for the clinical application and research of balanced translocation.

The limitations of this study include its small sample size, as it was an initial attempt to locate the precise breakpoints of balanced translocation with nanopore sequencing. The findings of this study are useful for genetic counseling and PGT. Hundreds of patients are diagnosed as apparently balanced translocation with chromosomal karyotype at our center every year. Approximately half of the seemingly balanced translocations were in fact unbalanced (De Gregori et al., 2007; Costa et al., 2022). The number of patients facing reproductive difficulty was large. The offspring of a balanced translocation carrier is likely to obtain a derived abnormal chromosome that leads to an increase (partial trisomy) or decrease (partial monosomy) of translocated fragments. The fetus will have an imbalance of genetic material, and the total amount of genetic material will be incorrect, resulting in fetal malformations or spontaneous abortion. In future studies, larger-scale samples will be recruited. At present, this patient is seeking assisted reproduction at the reproduction center of our hospital. By the time our manuscript was submitted, they had not started embryo transfer. By providing the precise breakpoints for them, we hope the patient will readily obtain normal embryos and block the transmission of chromosomal abnormity to offspring. In the future, we will continue to follow up on this patient until they give birth successfully.

## Data Availability

The datasets for this article are not publicly available due to concerns regarding participant/patient anonymity. Requests to access the datasets should be directed to the corresponding author.
