# Supplementary material for: Detection of a Cryptic 25 bp Deletion and a 269 Kb Microduplication by Nanopore Sequencing in a Seemingly Balanced Translocation Involving the LMLN and LOC105378102 Genes
Source: Front Genet. 2022 Aug 26;13:883398. doi: 10.3389/fgene.2022.883398 (PMC9469083; doi:10.3389/fgene.2022.883398)
Supplement: Supplementary file 1 [file DataSheet2.PDF]

Supplementary Material

Supplementary Table 1. False-positive breakpoints

Potential reads	Alignment location	Alignment location	Results
N1	chr3:186,504,197	chr6:158,703,972	False-positive
N2	chr3:181,628,700	chr6:166,529,664	False-positive
N3	chr3:197,798,186	chr6:145,320,011	False-positive

Chr, chromosome.

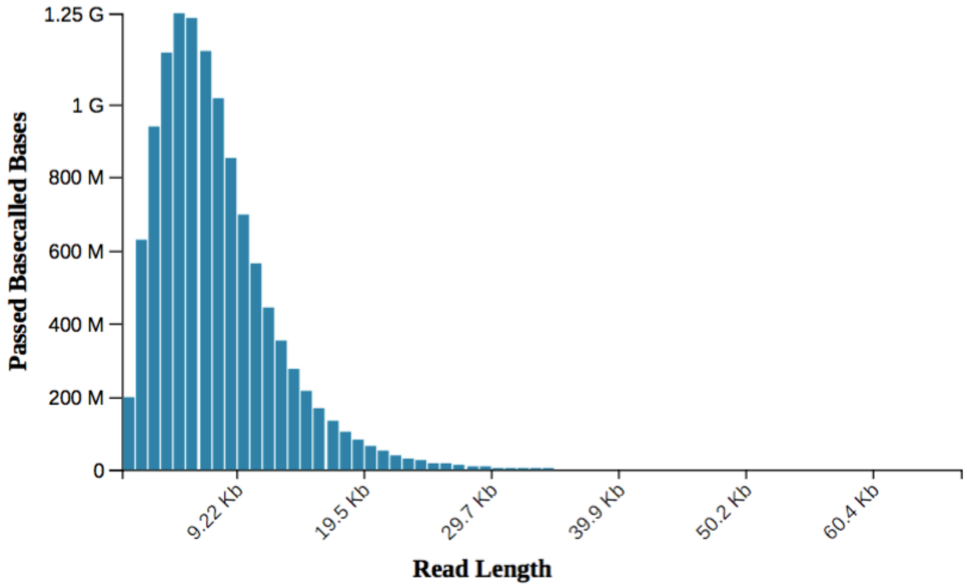

Supplementary Figure 1. Nanopore sequencing. Basecalled bases and read length based on nanopore GridION sequencer were shown in the histogram.

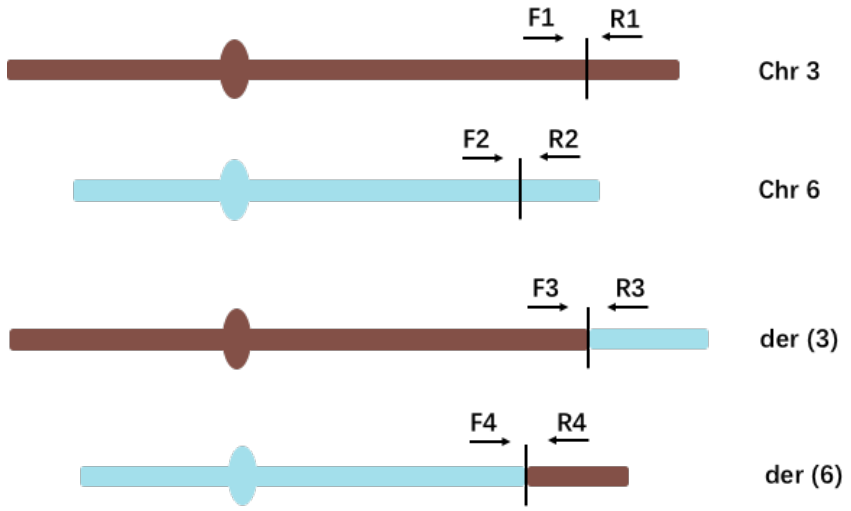

Supplementary Figure 2. Primer design. Primer pair F1/R1 was used for amplifying the normal chr 3, and primer pair F2/R2 was used for amplifying the normal chr 6. Primer pair F3/R3 was used for amplifying the der (3), and primer pair F4/R4 was used for amplifying the der (6).

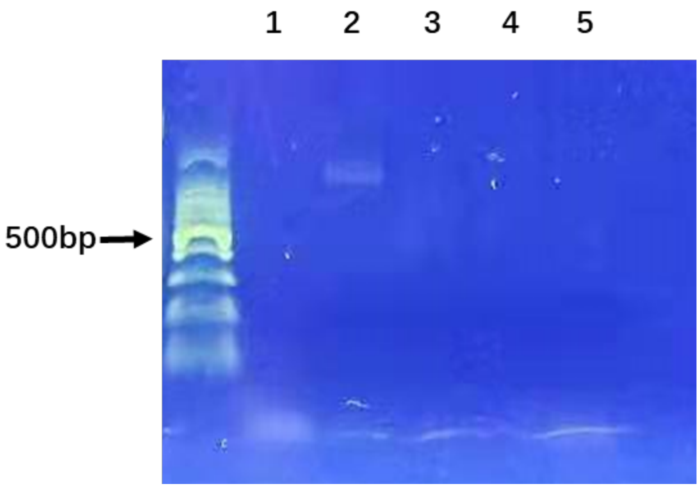

Supplementary Figure 3. Lanes 1 and 2 for N1 with two different primer pairs (F5+R5, F6+R6), lan e 3 for N2 with a primer pair (F7+R7), and lane 4 and 5 for N3 with two primer pairs (F8+R8, F9+R 9). The primers were as follows. F5: GCAGGAGTAACACCACAAT, R5: TCTTAGAGAATCTGAG GCTATC; F6: GCAGGAGTAACACCACAA, R6: GAATCTGAGGCTATCACACT; F7: GTCACAG ATAACTTCTCACTTG, R7: GGCTGGCTTGTTGAATGA; F8: CTAAGGCAGAAGGAACCATA, R8: ACTTACCTGACTCTACTAACTC; F9: AGGTAGGCACTGTCAACT, R9: ATTAGACTGGCG ATGATGAA. No expected PCR bands were visible, so these breakpoints were designated as false-po sitive.

Supplementary Table 2. CNV analysis

Variant type	Location and Length
dup(2)(q14.3)	(126682424-126802424)(0.12Mb)
dup(3)(q29)	(197733129-198013129)(0.28Mb)
del(6)(q16.1)	(94710282-94830282)(0.12Mb)
del(7)(q21.3-q22.1)	(98230688-98470688)(0.24Mb)
del(14)(q11.2)	(22272117-22511017)(0.24Mb)
dup(17)(p11.2)	(22373394-22640673)(0.24Mb)
del(17)(q21.31)	(45042632-45182633)(0.14Mb)
del(21)(q21.1-q21.2)	(22527681-22647681)(0.12Mb)

CNV, copy number variance; dup, duplication; del, deletion.

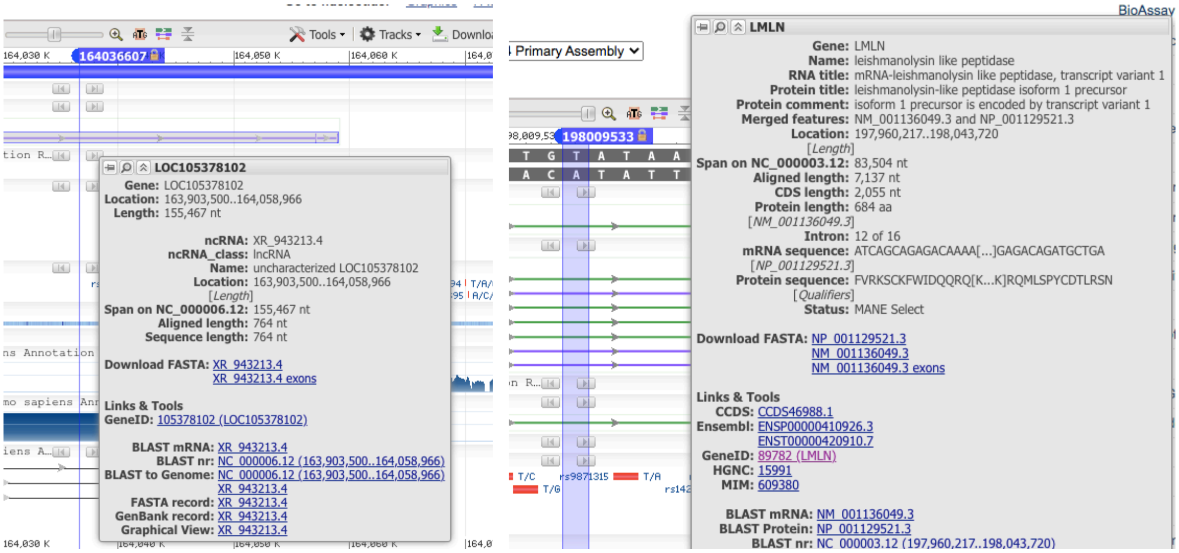

Supplementary Figure 4. The disruption of *LOC105378102* and the *LMLN* genes. *LOC105378102* gene is a lncRNA. *LMLN* gene includes 16 introns, and the breakpoint disrupts the intron 12 and leads to the early termination of translation.

Nucleotide sequences of three reads located at the breakpoints

Read 1 >89ba601d-4101-4f0f-bb18-0144917146ba

GTGTACTTCGTTCAGTTACGTATTGCTCTTGTCTTTTTTTATACTTTAAGTTTTAGGGTACATGTGCACAAC
ATGCAGGTTTTGTTACATATGTATACATGTGCCATGTTGGTGTGCTGCACCCATTAACTCGTCATTTAACGT
TAGGTATATCTCCTAATGCTATCCCTCCCCCTCACAACCCCACAGGCCCAGTTGACGGCTTGATGTTCCCC
CTTCCTGTCCATGTGTTCTTATTGTTCAATTCCACCTATGAGTGAGAAGATCGGTGTTTGGTTTTTTGTCCT

TGCCATAGTTTGCTGAGAATGGTGGTTTCCAGCTTCATCCATGTCCCTACAAGGGACATGGGCACTATCAT
TTTTATGGCTGCATAGTATCCCATGGTGTATATGTGCCACATTTTCTTAATCAGTCTATCATTGTTGGACAT
TTGGGTTGGTTCCAAGTCTTTGCTATTGTGAATAGTGCTGCAATAAACATACTTGTGCATGTGTCTTTATAG
CAGCATGATTTATAATCCTTTGGTGTATATACCCAGTAATGGGATGGCTGGGTCAGATGAACATTTCTCGTT
CTAGACCCCTGAGGAATCGCCACACTGACTTCCACAGTGGTTAAACTGGTTTACAGTCCCACCAACAGTGT
AAAAGTGTTCTTATTTCTGAACATCCTCTCCAGCACCTGTTGTTCCTGACTTTTTAATGATCGCCATTCTAA
CTGGTGTGAGATGGTATCTCATTGTGGTTTGATTTGCATTTCTCTGATGGCCAGTGATGATGAGCATTTTTT
CATGTGTCTTTTGGCTGCATAAATGTCTTCTTTTGAGAAGTGTCTGTTCATATCCTTCACCCACTTGTTGAT
GAGGTTGTTTGTTTTTTTCTTGTAAATTTGTTTGAGTTCATTGTAGATTCTGGATATTAGCCCTTTGTCAGAT
GAGTAGATTGCAAAAATTTTCTCCCATTCTGAGTTGCCTGTTCACTCTGATGGCTGTTTTTCTTTAGCTGCG
GAAGCTCTTGAGTTGGTCAGTCCATTTGTCAATTTTTGGCTTTTGTTGCCTTTGCTTTTGGTGTTTTAGACA
TGAAGTCACTTGCACGCCTATGTCACGAATGGTATTGCCTGAGTTTTCTTCTAGGGTTTTTATGGTTTTAGA
TTGACATTTAAGTCTTTAATCCATCTTGAATTAATTTTTGTATAAGGTGTAAGGAAGGGATCCAGTTCAGCT
TTCTACATATGGCTAGCCAGTTTTCCCAGCACGATTAAATAGGGAATTGTTTCCCCATTTCTTGTTTTTGTC
AGGTTTGTCGAATCAGATAGTTGTAGATATGGGACATTATTTCTGAGGGCTCTATTCTGTTCCATTGGTCTA
TATCTCTGTTTTGGTACTAGTACCATGCTGTTTTGGTTACTGTAGCCTTATATAGTATAATTTGAAGTCAGG
TAGCGTGATGCCTCCAGCACATTCTTTTGGCCTAGGATTGACTTGGCAATGCGGGCTCTTTTTAGTTCCATA
TGAACTTTAAAGTAGTTTTTCCAATTCTGTGAAGAGACCATTGGTAGCTTGATGGGGATGGCATTGAATCTA
TATGAGAATTACCTTGGGCATTATGGCCATTTTCACAATATTGATTCTTCCTACCCATGAGCATGGAATGTT
TTTCCATTTGTTTGTATCCTCTTATATTTCAGTGAGCAGTGGTTTGTGGTTCTCCTTGAAGAGAGGTCCTTC
ACATCCCTTGTAAGTTGGATTCCTAGGTATTTATTCTCTTTGAAGCAATTGTGATTGGGAGTTCACTCATGA
TTTGGCTCTCTGTCTGTTATTGGTGTATAAGAATGCTTGTGATTTTTGCATTCGTTTTGTATCCTGAGACTTT
ATGAAGTTGCTTATCAACAGAAATTTTGGGCTGAAGGCGATGGGGTTTCTAGATACAATCACGTCACCAAA
CAGGGACAATTTGACTTCCTCTTTTCCTAATGGAATACCCTTTATTTCCTTCCATCTGCTGATTGCCCTGCT
TCCAGAACTTCAACACTATGTTGAATAGGAGTGGTGAGAGAGGGCGTCCTGTCTTGTGCCAGTTTTCAAAG
GAATGCTTTAGTTTTGCCCATTCAGTATGATATTGGCTGTGGGTTTGTCATAGATAGCTCTTATTATTTTGA
GATACATCCCATCAATACCTAATTTATTGAGTTTTAGCATAAAGGGCTGTTGAATTTTGTCAAGCCTTTTCT
GCACCTATTGAGATAATCATGTGGTTTTTATCGTGGTTCTGTGTATATGCTGGATTACGTTTACTGATTTGC
ATATGTTTGAACCAACGTTGCATCAGAGGATGAAGCCCACTTGATCATGGTGGATAAGCTTTTTGATGTGT
GCTGCTGGGTTCGTTTTTCTTTATAAATTACCCAGTCTCAGGTATTTCTTTTTATAGCAACATGAGAATAGA
CAATATAATCTTACTAACTTCTTGAAACTCCCCATAATAGAAGCATTGTTGTTTTTTATTCAAGAGACAATG
GAAACAAGGATAACAGAATTCTAGGTTTCCAAGGTAGATGCAAGTGAGCTGGAACTGGTTTTCAAACTCTT
TTTGCTTCTCTGTAGCTTTTTAATTTCTCTTCCCTTGGTTTCCCTTATCTCCTTTTCCTCCTCAAATATGATC
TAATTATCTTAAAGCTGTACTTTAATAAAACCATTCTGAATTCTTCAGCTCCTTTGATTTTCCCCTTCTCTGG
CAGCTTACATCATTTGGAGTCATCACTGTATCCAGTCTCTGTTTCACATGAAAATGACCATTTCAATCCTCA
TAGTAGATAAGCTCACATAACCCTCACTTTGTGTGTCCTGTGATGGACATTGTGGCCCTTGCATTAAGTTAT
TACTGTGCCCTTATAAGACACGTGATATCAACAAGGAGAAACCAGAAGAGATGAGTTTAAAATGAGAGTGA
GAGCAGATTTCTTGGGCATCTAAGGAGCATAAGTCAAACTAGGATAGAGTGAACTTAATCTTTTTAGGAGC
CGAAAAATGATTCCAGACTGAAAGCTCACTTGGATCAACAGTCACAGCATGTAAGGCAGAGAAGGAACCA
ATGGGAATTGTCCTCAAAGTCTAAACTCTTTATTTTCTTTTTTTCTTCCTTCCTTCCTTCCTTCCTTCCACCT
TCCTTCCTTCCTTCCCTCTCTGTCTCTTTCTTTCTTTTGAGACAGAGTCTCACTGTGTTAAACAGGCTGGAG
GTCAATGGTGCAACTTAACAGCTCACTGCAGCCTTGACCTCCCGAGGGTTCAGCAGATCCTCCCACCTCAG
CGTCCCAGTATAGGGACTACAGATCTGCACCACCCTGTCCAGCTAACAGCCATTTTAAAATTTTTTGCAGA
GATGGGGGAGGGGGGTCTTACTATATTGCCCAGGCTGGTCTTGAATTCTTGGCCTCAGCTATCCTCCTGCC
TCAGCCTCCCAAAGTGCTGGGATGCTAAGTGTGAGCTACTAGGCCAAGCTGGATGTTCTTTTTACATGGTC
TAGTTTGTACTGGTCTAGTTACTTTAGGTAGGTCTAGTTTTGAAATTGTAGCAAGCTTGCTCCTCCCCAACG
CAACTATGTAGTAGGAAAAAATAAAGAATATTTTGACCTGGAGCTCTGTTGTGATTTTATCACACTTTGAGA
ATATTGAAGAATTAAGGGCCAGGCCTAGAAGGTGAGCCTGGACTCCAAACCCAAAGTGGTCAGGGTCAGG
CATCTTCATCCCTCCACTTGCCCTGTGAGAGCATTTTGTAATTAGCAGAATGCTGTGTGAGAACATGTAAG
ATACAATTCTTTACCATGTAATAATCAAAGTGCCACTATTTAGAATTTAGGTGTCAAAAAATATAGGATGCA
CTTAATGCAATCTTTCCGTATAATGTAATCAGAGTGTGAGGAGTGTCTTGCTCTATTAAAGAACAGTGAAG
CATGGGTCAAGACAGTCAAGCTGTTTTACTGCATCTAACTGTTCAGACTTCAGAAGGCAGGAATCCGTGGT
TGGATTTCATGAGTCACAGGTTTTATTACCTGTTCAACAGGTATTTTAAATAGCACTTGTTTTCCTTTCATG
TCAAACAGAAAATGAAATAAGCAGCTTTGAAAATGGTTACTTGGAGAGACAAAGGGAGATGTCCTTGATGT
CATCATTTAAATTGGTTCAAAGAGATGCTGAGGGCAAACAGAGCCACTTGTCGGAGTCACAGGGGGCAATA

GAGATGAGGCTGTCGCCTGAATGCTGTCCTTGTAGGACAGAATTCAGTTACAGGGCAAGCCCTCACTGAAT
GGGCTAAGTAGACTGCCATTCGACCTCAGGCAGCTGAGCTCTGTGTTTTAACACACTGCCTCTTTTAATGG
GGTCCCTGGAGAATGTCTTCATGGCTGACAAAAGCTAAATTGTAGCACACTCTACAGGGGCGCCCCATTTA
ATCTCCCTGTGTGATGTAGAGAAATCCATAGGCATTTTGTAGTATAATTGGCAAAATTTGAACATGAACCAT
AATTGGATAATAGTACAGCATCAATGTTAAATTTTCTAAACTTGGTAATTTTAACATGGTTATGTTAAGGAA
TATCCCTAGAAATATATTCTCTTTCTTTTTTTTGAGACAGAGTCTTGCTGTGTTGCCCAGGCTGGAGTGCAG
TGGCACAATCTCAGCTCACTGCAACCTGTGCCTCCTGGGTTCAAGCAATTCTCCTGCCTCAGCCTCCCGAG
TAGCTGGGATTACAGGTGCCCAACACCACGCCCAGTTAGTTTTTATATTTTTAGTAAGCAATACGTAGCA

Read 2 >cc814597-c09d-4135-9089-9242cdccb1e2

TGCTACGTATTGCTCCCTGAGCCTATGTTTTTATTTACTGTTTTGATAATGCTAAAGGAACAAAATATAATA
CACACAGGTAGTAGACTATTTGTATAATTTTATAACTTTTGCAAGCAAATCTTGTCTCTAAAAATAAAATAA
AAACTAAAACAATTGATTGCTTTCAATTAAATTTGTCTGGAAACTCCATCAGTGCTTTCTTTTTCTCTTACT
AGAACTTTTTTTTTTGAGACAAGGTCTCACTCTATTTCCCAGGCTGGAGTGCAGTGGCAGGCTGAGGTGAT
CCACAGCCTCAGCCTTCTCAGGTAACTGAGACTCATAAGTACACACCACTGCACACCTGGCTAACTTTTGT
ATTTTTAGTAGAGACAGGGTCTCACCATGTTACAGGTTGGTCTCGAACTCCCAACCTCACCGGTTTGGCCA
CTGCCTCAGCCCCAGAGTGCTGGGGTTACAAATGTGAGTCACTGCGCCCAGCTAGGACATTTAAAAAACTA
ATGGACATAGGCTGGGCACAGTGGCTCATACCTGTAATCCCAGCACTTTGGCAGGCCAAGGTGGGTGGAT
CACCTGAGATCAGGAGTTTGAGACCAGTGCAGGCAAACATAGTGAAACCCCGTCTCTACTAAAAATGCAAA
AATTAGTCAGACATGGTGGTGCATGCCTGTAGTCCCAGCTAGTTGGGAGGCTGAGGAAGGAGAATCACTTG
AACCATGAGGCAGAGCTTGCAGTGAGCTGATCGTGCCACTGCACTCCAGCCTGGGTGACAGAGCAAGACC
CTGTCTCAAAATAAAATAAAGTGAAAATTACAATAGAAATTCTCAAGTACTTATGTCAAAGTATAAAAGTAT
TTAATACCTATCATGTTCGGTTATGGAATCCCTGTGTAGCAGTCCCTGTGCGGCAAGAACGTTGTGAGTGA
TCACTTAAGGTGATGGCCTCTGCCTCCAAAATCTTTCTTAAGGTCCTAATGTGCTCATTGTTGAGATTCTCA
ATTGTTATGCCAGAGTTGTTTTTGAAAGGACAGCAGTAAAAATTGCTTTGTTTGAATTATTCCACAGCTGGT
ATAAAGCAAATTACAGCATGGCTGAGAAGTTAGACTGGGGCTGAGGAATGGGCTGTGACTTTGTCAGGAAG
AGCTGTGATTCTGGATTGATCAAAGAGACAAAAGTAAGAATGCATTTCCTCACAGTGTCACTGATTACACA
GTTCCTTTCTGACTTTATTCTTTTTTATCCTGAGCCTAAAATGCTATTTTCAAATGTAGTCTGAGCAGTTCAA
TTTTAAAATATATAGAAAACCATCTATAGATGGTTGGCAATAATATAGGAGAAGTTAGCTGTAAGTAGTTTT
TAATCATATACTAAAGGAATCCACAGAAGGGCCCACAGCCATGAACATTTCTTATTCCAGCTTTATTAGAA
CTCATAAATATATATATTTTACAAACCTTTGTTTCCTCTGTCATTTGTGGTGTTTTCTTCCAGAGAATGCACT
GTAAACATAGATGCAAATATTGATTCAGTATCAAAAAAAGGCACTTTTGAATAGGGGTTTCCAAATCCAGA
TGCATATTATAATCACCTAGCAAGCTTTTTAAAAAATACAGAGTAAGTTTGATATTCATCTATATATCATCT
ATACAGATACATATCACACATAGTAAGTTTCTGATATTCATATACCTATACAGATACATATCATACGTAGTA
AGTTTGACATCTATCTATATATCATCTATACAGATACATATCATACGTAGTAAGTTTGACATCTATCTATAT
CATCTATACAGATACATATCATACGTGAGTAAGTTTGCCATCTATCTATATATCATCTATACAGATACATAT
CATGCGTAGTGTTTCCACATCTATCTATATATCATCTATACAGATACATATCATACGTAGTAAGTTTGCCAT
CTATCTATATATCATCTATACAGATACATATCATACGTAGTAAGTTTGCCATCTACCTATATCATCTATACA
GATACATATCATAATGTAGTAAGTTTAAGTATCTATCTATATATCATCTATACAGATACATATCACATTTAG
TAAGTTTGACATCTATCTATATCATCTATAATAGATACATATCATACGTGTGTAAGTTTGCCATCTATCTAT
ATATCATCTATACAGATACATATCATACGTAGTAAGTTTGCCATCTATCTATATCATCTATACAGATACATA
TCATACGAGGTAGTTTGCCATCTATCTATATATCATCTATACAGATACATATCATACGTAGTAAGTTTGACA
TCTATCATATATCATCTATACAGATACATATCATACGTGAGGTTTGCCATCTATCTATATCATCTATACAGA
TACACAATACATTTATAAGTTCTACATCTATCTATATATCATCTATACAGATACATCACACGATAAGTTTGC
CATCTATCTATATATCATCTATACAGATACATCATACGTAGTAAGTTTGCCATCTACTTATATATCATCTAT
ACAGATACATATCATACGTAGTAAGTTTGCCATCTATCTATATATCATCTATACAGATACATATCATAATGT
GAGGTAAGTTTGACATCTACATATCATCTATACAGATACATATCATATACGTAGTAAGTTTGACATCTATCT
ATATATCATCTATACAGATACATATCATACGTAGTAAGTTTGACATCTATCTATATATCATCTATACAGACA
TATATCATACGTAGTAAGTTTGACATCTATCTATATATCATCTATATGTGGATACATCATACGTAGTAAGTT
TGACATCTATCTATATATCATCTATACAGATACATAATACATTTAAAGTTTGACATTCATCTATATCATCTAT
ACAGATACATATCATACGTAGTGTTTGACATCTATCTATATATCATCTATACAGGCTAAGTAACATACGTAG
TAAGTTTGACATCTATCTATATATATATCATCTATACAGATACATATCATTTATAAGTTTAGATTATTCATCT
ATATATCATCTATACAGATACATATCATACGTATAAGTTTGACATCTATCTATATATCATCTATACAGATAT
ATCATACATGTATATATACAGTTGTTCTTTAACCATGAGGATTGCTCATGGACCTCCCCTGATAAATAAAAC
TATAGATAGTTATGTAAAAGTTTCTTATATAAAATGGAATAGTATTTGCATATAACCTATGTATATCCTCCT

GTATACTTTAAATTACTTCTGCTATTTATAATACATAATACAATATAAATGCTATGGAAATAGTTGTTATAAT
ATATTGTTGTGGAAATGACAAAGAAGAAAAGTGCCCATGTTCAGCATACAGATTAAATCCATCATTTTTTCC
CTGAATTTTTCTTTTCTTTTTCTTTTCTTTTTTTTTTTTTTTTGAGACAGAGTCTCACTCTGTTGTCTAGGCTA
GAGTGCAGTGGCGCAGTCTCAGCTCACTGCAACCTCTTACCTACTAGGTTCAAGCAATTCTCATGCCTCAG
CCTCCCTGAATAGCTGTGATTATAGGCGTGCGCCACCACCCCGGCTAATTTTGTAAATTTTACTAGAGATA
GATTTTTCCCTGCTGGCCAGGCTGGCTCACACCTGTAATCCCAGCACTTTGGGAGGCCGAGGCGGGTGGGT
CACTTGAGGTCAGGAGTTTAAGACCAGCCTGGCCAACATAGTGAAACCCCATCTCTACCAAAAATATAAAA
ATTAGCCAGGCGTGGTGGTGGGCGCCTGTAATCCTAGCTACTCGGGAGGCTGAGGCAAGGAAATGCTTGA
ATCCAGGAGGCAGGGGTTGCAGTGAGCCAAGATCGCACCACTGTATTCCAGCCTGGGTGACAGAGTGAGG
CTCTGTCTCAAAAAAAGAGGGAAAAGCACACACCAACAAAATTGTTACATGAATTAATTCATATTGTATGT
GAACTTTTGAGCGCATTTTTTTACTCAGCATAATTCTCTGGAAATTCATCCAGGTTGTTGTATGTATCAATA
ATTTCACTTTTCACATGGTTAATATTCCGCAGTATGATACACTACAGTTTGTTTAAACATTCACACTTTGAA
GGACATCTGGATTGTTTCAGTTTTGGCCATTAAGAATGAAGTTGCTGTGAACATTTATGTACAGGTTTGTGT
GTGAACATATATCTTTATTTCCTCCAGAAAAAGTGCCAGAATGCAAAAGTTACTGGCTCATTACAATTACAG
GTTTAGTTTTGTAAGAAAATGCCAACTTTTTTAATTCCTGACAGACGTGTGCGATATATTCTTCCACATCTC
TTGTTGCATTTGGCGTTGTCACAATTTTTTGTTTAATCCGATTCTGATAGAGCCAGAGGATATTTATTATTTT
GGTTTTAATTTGTGTTTCCCTAACTGCTAATGATGTTGAACCTCTTTTCATGTGCTTACTCACTGATAATAT
ATCCTCTTTGGCAAATTGTCTCTTTGTCTTTTTTTTTCTGAGATGGAGTTTTGCTCACACCCAGGCTGGAGT
GCAGTGGCGCGATCTTGGCTCACTGTAACCTCTGCCTCCCAGGTTCAAGCAATTCTCTTTGCCTCAGCCTC
CAGAGTAGCTGGGACTACAGGCACGCATCTACTCATGTCTGGCTAATTTTTGAATTTTTAGTAGAGATGGG
GTTTTACCATGTTGGCCAGGCTGGTCTCGAACTCCTGACCTCGTGATCCACCTGCCTCAGCCTCCCAAAGT
GCTGGGATTATAGGCGTGAGCCACTCTTAAGCCAATTTTTTAATTGGATTGTTTTTTACTGTTGAGTTTTGA
GAGTTCTCTGCGTATTTTTGGGTATTTTGGGTAGTAGTAGTCTTGTCAGATATGTGGTTTGCAAATATTTTC
TCCTAAAGTACATGGGCTGTCTTTTTGTCCTTTTAACAGGGGCTTTTGTAGGGCAATGTTTTTAGTTTTGAC
GAGTCCAGAATATCAGTTTGTCCTCTTACTGTGTCTTTGATGTCAAGTCTAAGAAGTGTTTGCCAAGCTCTA
GATCTTGAAAGTTTTCATGTTTTTTTCAAAAGTTTTGTGGCTTATAATTTTACATTTAAGTCCATGATTCATT
TGAGTTAAGTTTTGTATAAGGTGGGAGACTTAGATCAAGGTTTGTTTCTTTGTGTACAGATAGGCAATGGCT
TTAGCACCATTTGTTGAAAAGGCTATCTTTCCTTCATGGAATTGCTTTGTGCCTTTGGCAGAAACCATCAGT
TGGGTGTATTTGTGGTGATCTGTTTCTATCTGTTTCTGTGTTTTCCGTTCTGTTCTAGCATCCATGTCCATG
CCTTCACCAATAACACACAGTCCTTAATTACTGTAATTATTTAATAAGTCTTGAAATTGGGTAGACTGATTC
TTTCCACTATTTTAAATTGTTCTGGCTGTTTTGGTTCCTTTTGCATTTCCATCTATGTTTTACAATTATCTCG
TCTGTATCTGTTAAAAGACTTGCAGTGATTTTGCTAGGAGCTGCATTAACTCCTGTGTGTCAGTTTGAGGAG
AATTGACATTTTTACTGTGTTGAGTTTTCAACTATAAACATGGGAGAAAATATTCAGTCTTCATTATTAAAT
ATAATGTTAGCTGCAGATTTTTTATACATGTTCTTTATCAAGTTGAGAAAGTAGCTAGCTCATTCCTGTGTT
TCTGAGAGCTTTAATCATGAATGGATATTGCTCTCTGTCAAATGCTCTTTCTACTTTAGCTGCTATGCATAA
TCATGATTTTTTCTACTTCTGTTAATATGTGACTTCTAGTTGATTTTCAAATATTAAATCACCCTTAGAGCTC
CTGGAGTAAACCCACTTGGTGACAGTAGCTTATAATTATTTCTGTATGTTGCTGAATTCTATGTACTATTAT
TTTGTTGTGGATTTTTGTTTCTATATTTGTGACGAATATAGATCTGGAGTTTTCTCTTTTTCCCCAATTATTT
TACCTTTAATTAATCAGTATTATCAGAACAATTATATTATGTCAGTTAAACTTGATCTTTTCAAATCTGACA
CATTTGAAAACAGGTATATCCTTCCAGTAAGAGAGATTTAAAAATAATAGTAAGAAGAAAATTTATGAAAT
ACATAATAATTATTACTAAATGAGCAAATAATGGGCTCTCCTGTCATACTTAATATTGACTTGATTTAAATT
ATTATTTATGGCCAGACATGGTGGCTTACACTTGTAATCCTAGCACTTTGGGAGGCTGAGGCAGGCTGATT
GCTTGAGCCCAGGAGTTCAAGACTAGCCTGGACAACATGGCGAAAGCCCATCTCTACAACAAAATACAAAA
ATGAGGCGGGCATGGTGGCCCATGCCTGTAGTCCCAGCTACTCAGAAGGCTGAAGCAGGAGGATCACTTG
AGCCCAGAGCTCAAGGCTGCAGTGAGTCATGACTGCACCACTGCACTCCATCCTGGCATCTCTACATAAAT
AAATGATTTATATAATACTCTGGAAGAATAAATCCCAGCATATGATGAGGTGTTTATCATGCTTTATTTTTA
TTTTGATTCAAATTCAAAATATTTTCTCTTTTTTAATTCAAAAACTCTTGTGTCACTTTGAGTTCAAAATATT
TTTAACTTTCTCTTGAGATTCTTCTTTGACCACTGTATAATTTAGAAGTGCTTTGTTTAATTTCCAGCTATTT
TGAGATGTTCCAGCTATCTTTCTGTTACTGATTTTCAGTTTAATTCCATTGTTGCAAGAGGTTAATACGGTA
TGATTTCTCTTTTACATTTGTTAAGGTGTGTTTCATGGCCCAGAATGTGGTCTGTCTTGGTGAATGCACCAT
GTAAGCTTGAAAAATCTGTTGTCTGCTGTTTTTGGTTGAAGTAGTCTGTAGAGTCTGCTATATCCAGTTTGT
TAATGGTGCTGTTGAACTCAACTGTGTCCTTACTGATTTTCTGCTCTGCTGCATCTGTCCATTTCTGAGAAA
TGGATATTCAAATCTGCAGTGATAATAATGGATCTAATTCATTTACCTTGCAGTTCTATCCATTTTGACTTA
CATATTTTGACACTTTATAGTTAGGTGCATACATGTTTAGGATTGTTATATTTTCATAGAGTACTGATTCCTT
GTCATTTTGTAATTCCTCTCTTATCTCTGATCATTTTCTTGAAAAGAAAAAGTTCTTTTTTGTACCATCTTTG

TCTAGTTTCTGTATCAGAGTAATACTAGCTTTATAAAATGAACTAGAAGTTTTTCCTCCTCTATTTTTCTGG
AAGAGATTGTTTTTACCAGACAGCAAAGATTTCAGAGGAAACTGTTATAATTGGCATTTCTGAATAATGCTG
GGGGAAGGGAACAATCACCAGGAATGTGGTGAATTTGTCAACGATCTCTATGATATCAAGGCGTGCAGAAA
GCTGAGATTTCATTTTTAGTATTGATTAAAGCTCTACCCAGGTATTATTAAAAGGAAAGGAAATTTCTGAGG
GAAAAGCTCCTTTTCTCATTGAGAAGTGTTTTAGGATACAGTAATAACATGAAAATGATGATGAGAACAGT
GGTAATAATAACAGCTAGAGAATTTATATTTATTTTTTTCTTTTCTTCTAAAATTTGATTATGGAAACCACAA
GACAGGAAGTTGAATTCCTTTTCTGGTAAGAATATATCAAACAGATGTCAAATAGATTTGATGCAGATTATA
ACAAAAAAAGGAAGGAATGCATATTAATTAAGCACTTACTGTGTGCTCAGTGCAGACAGATGTTTGACCTG
CGTTAATGTATTTGATTCTTTCCTGAATTTGGGATTGTAAAGGGGTTGTCAGAGTAGACAGTTATTAGTTAA
GATGGACTCAACATTGAAATATGCTTTTGCCTTTGTGGCTGCCTAGGAACCAGTGAAAAATAACGCATGTG
TCTTCCCTAGCATTGGGTCAAATTCAGCTGTTGCTTCTCACGCACCGACAGGATGCCGCAGCCCTCTTCAT
TCACCTCCTTTGAGACCACTGAGCGTGAGACAAGACTCAAAGTTAAGCAGAGCTCATCTGCAGGAAGGAAG
CCGCTGGAATCGCTGGTGGCCATAATCATCCCTTCTGACCATGAACTGGGCTTTTGATGAGTGTCGTTAGC
CTTGCTCAGAAGGAAAAAAAGAGGTTTTTTTTTTTTTTTTAAACCATGAACAGAGAAACATTTTACAGAAGT
CCACTTTTGGTAAATGATCTCCTGGATCAGCAAGTAATCTCGACATCTTCATATCATTTTTATACAAGATAA
AGCTGTTTGAAGAGTTTAAGATGAAAATTGGGTGAAGTTCTGCGTTGCTTTGTAATGACATATCCAGAGCC
ACTGCAATCCCTAGATAATGAGGAAATAATAGGAGGTGGGAGGCAGTAAAGGATTCCAAGATTTGTTTCTT
GTGTTCTTAGAAAAGACAGCCTTTTAGGAGATGGATGAGGAGCTCACTGTGGGCTTGCGGGGCTGGCAGG
AGCTGAGCTCCTCGAGGGGAAGCAGACTGGACCCCCTCCATGTGTGAACACAGAGACAGGCTTATTAGCA
CCAGGGCAGGTGGGTGCCACAGGACGTTTATTTTTAAATGAAACAGATTAAAATGTTTTCCCAGAAGAACA
GGCTTCTGAGCTAGGTTTATGGAGAGTGGCTCTGTGCAGCCAAAGGGGTTACAGACCCCTTCCCCACCCAC
CACCATGTGCGCCAGGCTGGTTTTCTGGGGCTCTGACTCCCAGGTAGAGGAGCTTCCATCGGTGGCTGCAG
GCCTGGGAGAGGTGGGCAGGGATGGGCCTAACGGTCTAAAGTCGAGGTGTAGAGAAACCCTGAAGGGGAG
TCTGAAGTGTTCTGCTGGTGACCCAGGGCAGGCAGATCAGTGGAGCAGTGGGAGGACAGAGATGGGGCCA
GACTCACTATTTCTGAGAGGTATTCCAAGCATTGAAATTCCTTCCTAATGAAGCTGGTACACCATTACTAAT
GAGATACTTTTATTTTTGGGGGATAAATGGGGATCAGAAGAGCTCAACTAATACTAAGAAATTCAAAGCAA
ATTTCTTGGTCAACGTGGTGAGACCCCATCTCTACTAAAAATACAAAAATTAGTCGGGCATGGTGGCAGGC
GCCTGTATCCCAGCCACTCGGGAGACTGAGGGAATTGCTTGAACCCTGGAGGTGGAGGTTGCACTGTGCT
GAGATCGCGCCATCACACTCCAGCCTGGGTGACAGAGCGAGACTCCATCTTAAAAAAAGCAAATTTCTGTG
GTAGGTCCAGCTATGTAAATTGTGGGGCCCAATAAAAAAACAAAAATACAGGGCCTGCCTGTTGTTCAAAA
AGCAGAAGGAAAGTACCATTACAGGTACTAAAATATGCATTTTTCTGTTTAAAAATATTTTATTACTTATAA
ACATAATACTAACAGTGATACATGAGTAACAGCATGAACTTACAAATTGTGAAAAAATACTTTTGTTTCATA
GTTTTACAGAGCATGATAAGTAACAATACTTTGTTAATGTGCTGTTTTGATTGATCATATGATTTTTCCTGC
TCTATTTTTCTGGAACCTTATTTATTAGATCATTGAAATTATACTTTTAGTAACCTCATTTTCATTGATATAT
GATTGAGAATGTTATCATTTGCTCTGGAAAATGCAAGATCACACATAATTTTTTATTTTAATTTTGAGAAGG
ATCTTCTCTGCTGATGCAATTATTATAGTAGCTGTTAAGAATATTTAGTAAGCTTTAACTCCATTGAGATAA
AATTTTTGATAAATCAATTCCAAAATACAAATTTTAGCATGGAGTTGATGATTATTCTCGGAACAATTTTTC
TAAAAGGATTTAACTTCTTCATACAGACTGTGGCTTTTGCAAATCTGAATTTAGTGTAAATTTGTAAAAACA
ATCATTTTAATGTCATATTCTTCTGATATTGTCTGTAATTGTGCAGTTGTTTGAGAAATGGATAGTGGCTTC
ATGACCTTGCAGTAATTTAAAAAATGCCTGTTTGCGGTGGCTCACGCCTGTAATCCCAGCACTTTGGGAGG
CCGAGGCGGGCTGGATCATGAGGTCAGGAGATCGAGACCATCCTGGCTAACACGGTGAAAGCCTGTCTCT
ACTAAAAATAGAAAAAAATTGCCAGGCGTGGTGGTGGGCGCCTGTAGTCCCAGCTACTCGGGAGGCTGAG
GTGGGAGAATGGCGTGAACCCAGGAGGCAGAGCTTGCAGTGAGCCGAGATCACGCCACTGCACTCCAGCC
TGGGCGACAGAGCGATACTCTGTCTCAAAAAATAAATAAATAAATAAAAATAAAGAAGAAAACATTTGTCT
TCCTTATTCATAACTGGTTTAGCTGGCTGCATAGGAAAACAGTGTTCTTTTCCATCAAATAAACATATTTAA
ATTTAATTTCTAAGAAAGATTATTCGTATGTATATCTGTAGACATTTGTTTTGCAGTGTTGTGCTAGCTTTC
AAAAACTAGAGATTCAAAACTTTAAAGAGCTAAGCTTCATGGTGATGCCCACGCATGCACTTTTACTTTGT
AATAATCTACTGACAGGTCTCTTTGGGCTATTGGACTGGTAGTTCCTGTAGCGGAGCTCAGGTTGGAAAGC
CAGTGGCAGAGGCTGCTTTGCAAGGACGGGCTCCAGGTAGTGACGGCAACCAGCCCTCCACGTGTCCTGG
CTCTGTCCTCGCGCCAGCACCTCTCCTCTCCTGTGGCTGTGGCTGCCGCTATAGCTGCTATCTCTGCCACC
AGCCCGGGCAGGTCACAGCTCCTCCGCCCGGTGGAGGCCCCCGCACCGAGGCAGCACCAAGGATTGGTTC
TGGCTGTCTGCGCATGCGCTCACAGGGCTTGCGGGTTTCTGATCTGCGCATGCGCAGGCTCTGCTAGGCTA
GGGCCACCTGGTGCTTCCCACCACGCCCTGCAGGCGCCTAGCCTGGGTTCTCCTTCACTTGAAAAATTAAA
AGATAAGACTCATCCAAGACAAGGCAGCAGAGCATTCAGCCAAGCATAGGGGCCTGTGCCACTGTACTGC
ACTGATTGCACACCCAAGAAGCCAGCCCTATCAGGCAGGCAGGATCACCTGGGTGTCTGGAATACCCCCTT
TTGGAGAAACATATTCTTTAAGATGCAGAAAGCATGGAGAAGGACATCTGTGGAGGGCTTCCGAGTGGGGA

ATTATAAAAAGAGCGGCCGGAGAGCAACTGAAATGAAGAGACTATGTTCAGATACATGGCCTAGGAGCCA
CTGATGAATTACACCTTTTTTTTCCATAAACCACAATCACAATGTATTGTCAACACTTTCCACTTTGAGTTT
CTTAAAAGAGGGAGCTAGAAGGAAGATATTTCTAATGCCTTAAAGAAATTGAGCTTTCCCTTCCTTGACAT
TTTACACTTGCCTTTTCCTTACCTTATTTTAAAGCACCTATTTTTAGTGACTCAGCTTTGTAGTAATTTCTAG
AACATTCAATTTGCTCTCTGGTGAGTTGGTTAAATGGAAATTGGTTAAGAAAGATCTCTCCACTGTCTACAA
GGTGGGGGGACACATATTTATCTCCCAATGTCTTTTTATGAAGGTCTGTGCCCAATAACATTCACAGATGA
TTATTTTTAAGTAAACTTAAAAGATGAACAACTGAGTTAAATAGTCCCTCTCTTGTTCTATCTTTGATGATT
CAGACATCTTTCCTCTTCATTCTTTCTCATTCTACTTAACTTCCGAATGCTTAGAAGCTCATCTATATGGGA
GAGTGAGATGCATATGAAACGGCTTTTCTGTTGGAAGAGTGCCTCACTAAGCCTGTGACGGCCATCCTGTG
TGTGTGCCTGATCTGAGGACTTTACATTAAAATAATTTAATTTGGTGATGGTATTACCTGGCATTTGTTTGA
GCTGAGGATTACAGGCTCTTCTTCTTCTAGCTAGTTCAAATTTTATTCTTGAACCAAAACCAGCAGGTGAGT
ATTTAGAGTGCATTGTTTTAGTTCATAGTTGACTTGTTCCTATCAAATGGTGCTTTGGTGTCATGTCATTGG
TACAAATTGATTGACCTCAAAAGAAATACTAGCAAACTATTTTTGCATTTATTATAGAGATTTCGGGGACAT
CGCTCAATAAATCCTAAGTGTCTTTTTTTTATGAGGGTCCAAAATTTAAATCTCTTTATCATCATAGTGTTA
GAAAAAAAGTTGCATTTATTTCAAGCAACTATTTCTTTGTATCTTGTCTGCTAGGATACTCAGACACATCTT
CTACCACCCTCTTTTTTTTGAGATGGAGTCTCACTCTGTTGCCCAGGCTGGAGTGCAGGGGCATGGATCTC
ACCTCACTCCAACCTTGCCTCCCAAGGTTCAAGTGATCCTCCTGCCTCAGCCTCTGAGTAGCTGGGATTAC
AGGTATATGTGCCACCATGCCTGGTGAAGCTCCACTATCTTATAATGACTGCAGAAATACAAGGAATGAAT
ACATTGACTCTCCACTTTCTTTTTTTCCCTACTTGTTTTTTTTCTTTACTCTGGGTTTATGTTTATTTTTAAAA
CTCTTATGACATTTGTGTAATGATATAAATCATGTCAAACCATTGTTGTAATAGAGTGGAATTGAAACACAT
GAGCACAAAACCCTCTGAAGAACTCATAAACCCTTTTCATCTACTTACTTCATCTTCATGCTTAGTTGAATG
ATCGCCATTCCTAAGGAGAGGCCTGTTTTCCTGGATGTTGGCAACTTATACCCCTACAGCCAGATGGTACA
CATCTCAAAACATGGCCTTCAAACACTCTGCCTATGAAGCTGAGGAGAGGAAAGGAAAATTTATTGAGAGA
GTCATAGAAGCCAATCCACAGAGACAAAAGTATTGGTGTCATCAAATGCTGCACAGCCTAAAACCTCTGAG
GGCCCACGAGTCTGGCCTGGTGCCACAATAAGCCAACCATTACCGTAGGGAAATTCAGTGTGTCTGACAAC
ATTTCCCACTCTAGTTCATATGCTCATGGTGTTCTTGTGGGGCGGCCATCCAGGCCTCAAGCCAAAATGAG
GTTTCCCCACTGTGGCCACATCTCTTGATAGAATTCAGAGGTTCTGATAAGGTGCATATCTTCAGTCTGCA
GACGTCCTCCCTATGTCACTGTGGTCATGCAAAGTGCTATCAACGTATTATGGTTAACATTTTGTGAAGAAA
ACTTTTTGGATCATCATATTACAGGCCCTGCTGAGGCCTGGGCCCAAAGGCTTTGTATTTCAGCACAGCTA
GCTTGTTTTAATGCTCTATTTCCCAGCAGTTTCAAACTCACAAAGATGGCCAACAGTGGCAGATGGGACCT
GACGCTTGAACTTGGGGAGCTTTCCTCCAACAGGATGAATCCATGGAAGATACCAGGTACATTCTCAAAAG
AACAGAAGTTTAGCCAGGAAATGAGAGAGGGTGGATGCAAGTTAAAATTGTTGATAAGTAAATACCCTTGA
TTGGGTATTCCTGTCAGGTGCTGCTGTAAAAATCTCTTAAAACACTGGGTGAAGTGGATAAATGGATTCCA
TTTAACAGAGCATTTACTGGGGTCTATTAGCTGACTATTACCTAATGATTATATTATAGGCGGCAATGGGGC
TGTCATCATAAAAGGAAATGATCAAGGAGAAAGAAGTATGAGCCTGGAAGAGGATGCAGCAATGCATGAG
ACAGGAGTTGTGCCTGTTGGCCTCCTTCCAGTGAGAAGGCTGCATCTGAAATTATCACTGAAGTAGGAAAA
ACAGTGTCCTTATTCTTTTTGTTTATTCAAAAATGCAATAGCACACTGATGCATATGGAACCTAAATATGAG
AATTGTAAAAAAATTGACCTGGGATAGTTGTGTTGGGGTTATCAAACAGAATGTTGGTATTGGGAGGATCC
TGGGACACATGGAGGTGTTAGATCAATTGGGGACTGTGGACTTTTGTTTCAGGTGAACCTTTTGGAGGTGG
GTGTGAGGGGTTGGTAAAACTTGAACTTGACCTTCCAGTGAGTCCAAATGGCTTTGACAAACATGTGGTGG
GCAAGGCCTAAGAACAGAGAGTGAACAACTAGGAGCCCTAATGAAGTAGGAGAGTTAGATGCTCATGTCT
CGGAAAGCAAGACGTCGAGGAGCCTCTAAGCAGGTGGTCCATGGTCTCATGTGGCCAGAGACTCAGAGCA
GGGATGGCATCTTGAAGGAGCAGAGAAAAGCTGAATGGGTCATAGAGATACCGGCAGACAATGAGTGATG
TGTGCAAAGTTGCCAATACATGGTAGATATGGTTTGCTCTTGTGTCCCCCCAACTTACCCCATCTTAGCTCC
CATACCCCTACATGTTGGGGTGGGACCTGTGAGACGATTGGAATTATTAGGTGGGTCTTTTCCTGTGTTGT
TTTTGGTGGATAGTGAATGGGTCTCACGAGATCTGATGGTTTTAAAAATGGGAGTTTCCCTGTACAAGCTT
CTTCTCTTGCCGCCATGTAAGAAGTGCCTTTCTACCTTATTATGTTTTTGAGAGAGTCTCACTCTGTTACCC
AGACTATAGTGCAGTGACACAATCATAGTTCACTGCAGCCTCAACCTCCTAGGCTCAAGCGATCCTCCCAC
CTTAGCCTCCCAAGTAGTTGGGACTACAGGTATAGGCCACCACACCTGGCTAATTATTTTATTTTTTAGTTT
TTCATAGATGGGGGTTTTACTTTGTTTGTAATGTTGTTCAGTTCATTCCTGGAATGGAATCTTACAGATTGC
CTTCCCTTTTTTTTTTTGCTTTCCTAGAGAGACTTATACCTGTTTTTTACCAACCAAATTTTATAATTTTATGT
TATAATAATTTTTATCTTTTCACTTGAATTCATTTGTAAAGAAAAAAGACTTGAAGGAAATGCACTATACTA
TGTTATTAGTGATTATCCCTGAAGGGTTAGCATGATGGTGGCCTCTCCAGTCTTCTGTATTGTTTAAATTGT
CATCATAGAACACTTACTACTTTTATCATCAGATCTAATTGATGAATGCTATTTTTAAATTTTGTCTAAAAG
TGACTATTCCTCTAATATCTTTTTTAAAACCATTAAACTTTCAGCTTCCTTCAATGCATGTACATACCCTTGT

TGAAAAAAGCCATAAAAAATTAAAAAGCCTTCAGAGATTTGCTTATTTATACATGGCATTAGTGTCCACATT
ATACCAGAGATCACATACTCTCAGAATCATTCTGTACAAGTGCCTAGTTGACTACCTGTATTCAAATACCAA
GGAACAAAGTAAAATAAATTTCTATTCCTTTTTCATTTCTGTTTTCCATAGACAGTTTTATAAAGGTATAAG
CAGCTAAGAATATATGTATATTGTGCAAATACATAACATTTTTAGGATATATTTTTGTGTAGTCACCTTACA
AGTCTTTAAAGTATTTTTTCCAGCAATCCTGAATCAATTCTGTATGGGAACAAAAAGGAAGGAAAAATTTAG
GGGGAAGTGCTTTAATCCTTCATATGGTGATGCTCTGTAGCTCTGGCCTATTCTATAATCGGCTGTGTACTA
CATGTATCTTAGATAGGACTATTTATTGTGTTCATTGTTGGGGCAGTGGTAGCTGGCAGTTTCAGCAGCTAT
TCAAATGTCTTCATATTAGAAGCATCTCACATGGAGCTCATGAAGGTGGTCCTTTGCCTGGTGTATTAGTCC
CTTCTCACATTGCTATAACAAACTACCTGAGACTGGGTAATTTATAAAGAATAGAGGTTTAATTGACTCACA
GTTCCTCAGGCTGTACAGGAAGCATGGCTGGGGAGGCCTCAGGAAACTTACAATAGTGAAGGCAGAGAGG
AAGCAGGCATGTGCTACATCACTTGAGTAGGAGGAAGAGAGAGTGAAGGGGGGAGGTGCTACACATTTGA
ACAACCAGATCTTGTGTGATCTCACTCACCATCATGGGAACAGCAAAGGGAAATCCTCCCTATGATCCAGT
GACCTCCCCACCAGGCCTCCTCCAACACTGGGGATTCCAATTCCACCTGAGATCTGGGCAGGACACACATT
CAAATCCATATCACCCGCAAGTAGAAATATGCTAGTGTTTGACAGTATGACTATAGTGCAGAGGGACTTTC
AGTCATTAAAATCATGAAGGAGTCTACATAACTAGAAAGTAATTAAATTTTTTATAATTTGCATTTTAAGAA
ATTAAATGCCTGGAGAAGAAAAAAAGCAGTTTTCAATAATATATTAGAGTGATAGTATACTGGAGGTGAAG
GGAAGTTAGTTAATATTTAGTCAAATCTCCTTATTTTACGATTTTAAGGAAAAGGTTGAGAGTTAAGCAATG
GAAAAAGATTGCCAAAATTAGCATTACGATATAAAACTGAAGAAATTTAGGAAAGTATTCAAAAATTAAAG
AAAGTGTTTTGAGAAAATTTTTCAGAAAAGAACAGAAAAAAACATTTTAATATCCTGGTTGGTGCTGGTGG
GGGGTTATTTAAGAAATCTTTTTATTTCCCAAAGATCATAATGATATTCTGTTTTCTTTTTTCTTAGAAACAT
TGTTTCAAGATTTTATATTTAGGTCCTTGATTTTCTCAAATTATTACTCTATTTTTTGAGACAGAGTCTCACT
CTGTTACCCAGACTATAGTACAGTAGCACAATCATAGCTCACCGCAGCCTCTAACTCCTAGCTCCAGCAAT
CCTCCCACTTTGGCCCCCTCAAGTAGCTAGGACTACAGGTACAGGCCACCACACCTGGCTAATTATTTTAT
TTTTTAATTGCTTCTGTAGATGGGGGTCTTACTGTGTTACCCAGGCTGGTCTTGAGTTCCTGACCTCAAGTG
CTCTCCCACCTTGGCCTCCTAAGTGCTGAGATTACAGGGGTGAGCCACCACTCCCAGCCTTAAATTAATTT
TTGTGTGGTGTGCTAGGGGGATTGCCATTTTATTTTACATTGAATTAATGATTTCACGGCTGAAAAGATCAT
CTCTTCTGTAATGAATTGCAGTGGAGTCTGTTTCATAAATCATGTGATGTGTATAAATTTGAGACTGTTTTT
GACACTGCTTTGTCTATTACATTGGTCTATTTATCCAGTTTCGCCCCAGCATAGCACTGTATTGTTACAAGG
GCTATACATCTCCAGCCTTATTCTTCTACATTAGCATTGGCTTGGCTATCCTAGTTCCTTCCAATTAATATA
TAAATTTTAAAATCAGCTTGCCAATTTCTATAAAATAAAATATTGTGGAATTCTGATTAGGATTGCATTGAA
TCTTGGGTTCAAATATCCAAAGATTTCTATTTATTTGTTATTACTTGCTTTTGCACTACAATTATTTATTTGC
CATAGTTTGTATGTCATGTGTCTGATAACTGATCGCACATGTAAGACAACTGGCCATCCACTTGGACATGTT
TAAATTTTTTTACTGGCCGCATTGAGGTAGACTTGACAAAATTATATATATTTAAGGTGTGCAACATAGTGT
TTTGATATAGGCATACATTGTGAAATGATTACCATAATTAAGCTAATTATGTATTTGTATATATCTATATCT
AGATAACCTTATATAGTTACAACTTTTCGATTGCAGTGGTAAGAACACTTTAGATCAACTCTTAGCAAATTT
CAAGTATGTAGTACAGTATTATTAACTATAGTCACTATGCTGTATATTAGATCTCCAGAATTTATTCTTCCC
ACCTAACTGAATCTTTGTATTCTTTAAACAACATTGATTTATTCAATCCATGAATGTGGTATGTCTTTTTCTC
TTATTTAGATAATCATTTCTTTCTTAGCTGTGTTTTTGAGGATTTAGTGTTGATGTCATGGACATTTTATTCA
AATTATTTAATTTTTGATGCGATTGTAAATGGTATTTAAAATTTTACTTTCCACATGTTTATGGTTACTTATA
CAGAAATATATACATTTTTGCTAATTGACCTTTTATTCAACAACCTTCTATTCATATCATTTTTATTAAATTG
CTATAGATTCTTTTAACTTTCTACATGTATAAGTTTGCTGGGGTGTAGGCTCCTCATGGAGAGCCTCATAGG
GTAGGGTGGAAGGGAAATGTGGGGTTCGAGCCCCCATCCAGAGTCCCAACTTGAGCACTGCCTAGTGGAG
CTGTGAGGTTAGCGGCCACCATCCTCCAGACCCCAGAATGGTAGATCCACTAATAGCTTGCATCATGCTCC
TGGAAAGCTGCAGACACTCAACACCAGCTTGTGAAAGCAGCCAGGAGTGGGGCTATACCCTGCAAAGCCA
CAGGGGCAGAGCTTCCTGGCTGTGGAACCCCACCTCCTGCATCAGTGTGACCTGCATGTTAGACATGGAGT
CAAAGGAGATCATTTTGGAGCTTTATGATTTGATTGCCCTGCTGGATTTTGACTTGCACAGGACTTGTAGCC
CCTTTGTTTTGAGTACGTAACTGAACGAAGTACAAT

Read 3 >a8402eed-6219-4c6a-bfd6-10cc72f44706

TAAGCCATTGCTGATCATTTTCTTGAAAAGAAAAAAGTTCTTTTTTGTACCATCTTTGTCTAGTTTCTGTATC
GAGTAATACTAGCTTTATAAATGAACTAGGAAGTTTTTCCTCCTCTATTTTTTCTGAAGAGATTGTTTTTAC
CAGACAGCAAGATTTCAGAGGAAACTGTTATAATTGGCATTTCTGAATAATGCTGGGGAAGGAAACAATCA
CTCAGGGAATGTGGTGAATTTGTCAACGACTCTATGATATCAAGGCGTGCAGAAAGCTGAGATTTCATTTT
TAGTATTGATTAAAAGCTCTACCCAGGTATTATTAAAAGGAAAGGAAATTCTGAGGGAAAAGCTCCTTTTC
TCATTGAGAAGTGTTTTAGGATACAGTATTAACATGAAAATGATGATGAGAACAGTAGTAATAATAACAGC

TAGGAGAATTTATATTTATTTTTTCTTTCTTCAAAATTTGATTATGGAAAACCACAAGACAGGCTGAATTCT
TTTCTGGTAAGAATATATCAAATAGATGTCAAATAGATTTGATGCAGATTATAACAAAAAGGAAGGGAATG
CATATTAATTAAGCACTTACTGTGCCTGTGTGCAGACAGATGTTTGACTCAGTGCGTTAATGTATTTGATCT
CTTCTGAATTTGGGATTGTAAAGGGGTTGTCAGAGAGACAGTTATTAGTTAAGATGGACCTCAACATTGAA
ATATGCTTTTGCCTTGTGGTTGCCCAGGAACCAGTGAAAACAAATCGCATGTGTCTTCCTAGCATTGGGTC
AAATTCAGCTGGGTGCTGCTTCTCACGCACCGGACAGGATGCCGTGTAGCCCTCTGTCTACCCCCTTGAGA
CCACTGAGCGTGAGACAAGACTCAAAGTTAAGCAGGAGTCTGATCTGCAGGAAGGAAGTCGCTGGACCAA
GCTGGTATTACAATCATCCCCTCTGACCATGAACTGGCTTTTGATGAGTGTCGTTAGCCTTTGTTCAGAAGG
AAAAAAAAAAGAGGTTTTTTTTTTTTTTTTTTTAAACCATGAACAGACAGAAACATTTACAGAAGTCCACTT
TTGGTAAATGATCTCCTGATCAGCAAGTAACTTCGACATCTTTCATATCATTTTTATACAAGATAAAGCTGT
TTGAAGAGTTTAAGATGAAAATTGGGTGAAGTTCTGCGTTAAGCTTAGTAATGACATATCCAGAGCCACTG
CAATCCTAGATAATGAGGAAATAATAGGAGTGGGTGGAGGTGCAGTAAAGGATTCCAAGATTTTGTTTTTG
TGTGTTCTAGAAAAGACAGCTCTTAGGGAGATGGATGAGAGCTCACTGTGGGCTTATGGGCTGGCAGGAGC
TGAGCCCTCGAGGGAAGCAGACTGGACCTCTCCATGTGTGAACACAGAGACAGGCTTATTAGCACCAGGG
CAGGTGGGTGCCACAGGACGTTTTATTTTTAAAATGAAACAGATTAAAATGTTTTCCCAGAAGAACAGGCT
TCGAGCTAGGTTTATGGAGAGTGGCTCTGGGTGCAGCCAAGGGGTTACAGACCCTTCCCCACCCACCACCA
TGTGCGCCAGGCTTTGTTTCGGGGCTCTGACTTCAGGTAGAGGAGCTTCCCATCGGTGGCTGCAGGCCTGG
GAGAGGTGGGCAGGGATGGGCCTAACGGTCTAAAGTCGAGGTGTAGAGAAAACCCTGGAAGGGGAGTCTG
AAGTGTTCTGCTGGTGACTATGGGCAGGCAGATCAGTGGAGCAGTGGGGAGGACAGAGATGGGGCCAGAC
TCACTCCTCCATTTCTGGAGAGGTATTCCAAGCATTGAAATTCCTTCCTAATGAAGCTTAATACACCATTAC
TAATGAGATACTTTATTTTGGGGGGATAAAATGGGGATCAGAAGAGCTTCAACTAATACTAAGAAATTCAA
AAAGCAAATTTCTTGGTCAACGTGGTGAGACCCCATCTCTACTAAAAATACAAAAATTAGTCGGGCATGGT
GGCAGGCGCCTGTATCCCAGCCACTCGGGAGACTGAGGGGAATTGCTTGAACCCTGGAGGTGGAGGTTGC
ACTGTGCTGAGATCACGCCATCACACTCCAGCCTGGGTGACAGAGCGAGACTCCATCTTAAAAAAAAAAAA
AAAGCAAATTTCTGTGGTAGGTCCAGCTATGTAAATTGTGGGGCCCAATAAAAAAATGAAAATACAGGGCC
TGCCTGTTGTTCACAGCAGAAGGAAAGTACCATTACAGGTACTAAAATATGCATTTTTCTGTTTAAAATATT
TTATTACTTATAAACATAATACTAACAGTGATACATGAGTAACAGCATGAACTTACAAATTGTGAAAATACT
TTTGTTTCATAGTTTTACAGAGCATGATAAGTAACAATACTTTGTTAATGTGCTGTTTTGATTGATCATATG
ATTTTTCCTGCTCTATTTTCTGAACCTTATTTATTAGATCATTGAAATTTATACTTTAGAACTTCATTTTCAT
TACATATGATTGGAATGCTATCATTTGCTCTTGAAAATGCAAGATCACACATAATTTTTTATTTTAATTTTG
AGAAGGATCTTTCTGCTGATGCAATTATTATAGTAGCTGTTAAGAATATTTAGTAAGCTAACCCATTGAGAT
ACTTGATATAAATCATCCCAAAATACAATTTTAAGTACGGAGTTGATGATTATCTTTGAACAATTTTTCTAA
AAGGATTAACTCTTCAGCGATCAGGTTTTTTGCAAATCTGAATTTGCAAATTTGTAAAATAACCATTTTAAT
GTCATTCTTCTGATATTGTCTGTAATGTGCTGTTTCGACAGAGTGGTGGAAATGACAGTGGTTTACAGATCT
GCATGCAATTTAAAAATGCCTGTTTGCGTGGTGGCTCACCTGTAATCCCAGCACTTTGGGAGGCCGAGGCG
GATCATGAGGTCAGGAGATCTGCACCATCCTGGCTAACACGGTAAGCTCCGTCTCTACTAAAAATAGAAAA
ATCAGCCAGGCGTGGTGGTGGGCGTCTGTAGTCCCAGCTACTCGGGAGGCTGAGGCAGGAGAATGGCGTG
AACCCAGGAGGCAGAGCTTGCAGTGAGCCGAGATCACACTGTGGCCTCAGCCTGGCCACAGAGCGATACT
CTGTCTCAAAAATAAATAAATAAATAAAAATAGCATGTTTCATTTGTCTTCCTTATTCATAACTGGTTTAGC
TGAAGTTTTTTGTATAGGAAAACAGTGTTCTTTTCCACTAAAATGTAAACATATTTCTAAATTTAATTTCTAA
GAAAGATTATTCGTGCTGTATATTCAGACATTTGTTTTGCAGTGTTGTGTTAGCTTTCAAACTAGAGATTCA
AAACTTTTAAAGAGTTTTCAAGCTTCATGGTGATGCCCACGCATGCACTTTTACTTTTGTAATAATCTACTG
ACAGGTCCTGCTGGCTGTTCATGTAGTTCCTGTAGCGGGAGCCCAGGCTGGTGCCAGTGGCAGAGGCTTTG
CGCAGACGGGCTCCAGGTAGTGACGGCAACCAGCCTCCAATGTCCTGGCTCTGTCCTCGCGCAGCACCTC
CTCTCCTGTGGCTGTGGCTGCCATAGCTGCCATCTCCGCCACCAGCCTGGGTGCAGGTCACAGCTCCTCCG
CCTGCAGGCCCCAGAACACTGAGGCAGCACCAAGGATTGGGTTCTGCTGTCTGCATGCGCCTTCACAGGG
CTTGTGGGCTTGATCTTGGCATGCGCAGGCCCTGCTAGGGTTAGGGCCACCTGGTGCTTCCCACCACGTCT
TGCAGGCGCCCAGCTCAGTTCTCCCTTCACTGCAAAATTCAAGTTAAAAGATAAGACTAGTTCAAGACAAG
GCAGCAGAGCATTCAAAGGCATAGGGGCCTGTGCCACTGCATTGCACTGATTGCACACCCAAGAAGCCAG
CCCTATCAGGCAGGCAGATCACTTCCAAGTGTCTGGAATAATTTTTGGAGAAACATATTCTTTTAAGTATGC
AGGTGGAAAGCATGGAAGGGACATCTGTGGAGGGCTTCCGAGTGGGGAATTATAAAAAGAGCGTTTGAGA
GCAACTGAAATGAAGAGACTATGTCTAGATACATGGCCTAGGAGCCACTGATAGAATTACACTTTTTTTTTT
TCCATAAACCACAATCACAATGTATTGTCAACACTTTCCACTTGAGTTTCTTAAAAGAGGAGCTAGAAGTTG
AAGATATTTCTAATGCCTAAAGAAATTGAGCTTTCTTTTCCTTCCCTGATATTTTAATAATTTATTTTTCCTT
ACCTTTATTTTAAAGCACCTATTTTTAGTGACTCAGCTTTGTAGTAATTTCTAGAACATTCAATTTGGTTTCT

GGTGAGTTGGTTAAAATGGAAATTGGTTAAGAAAGATTCTCCACTGTCTACAGTGGGGACACATATTTATC
TCCCAATGTCTTTTTATGAAGGTCTTGTGCCCAATAACACTTCATGATGATTATTTTTATAAATGAAGATGA
ACAACTGAGTTAAATAGTCCTCTTGTTCTATCTTTGATGATTCAGACATCTTTCCTCTTCATTCTTCTCATTC
TACTGCTATCTCCCGAATGCTTAGAAGCTCATCTATGGAGAGAAGAGATGCATATGAAACGGCTTTTCTTA
AGCTGGAAGAGTGCCCCACTCAAGCCTGTGACGTCATCCTGTGTGTGCCTGATCTGAGTGACTTTTACATT
AAAATAATTTAATTTGGTGATGGTATTACCTGGCATTTGTTTGAGCTGATGGATTACAGGCTCTTCTTCTTC
TAGCTAGTCTAAATTTATTTTGAACCAAACTGTGTGTGAGTATTTAGAGTGCATTGTTTTAGTTCATAGTTG
ACTTTTTTGTTCCTATCAAATGGTGCTTGGTGTCATGTCATTGGTACAAATTGATTGGACCAAAAAGAAATA
CTAGCAACTATTTCTTGCATTTATTATAGAGATTTTTTGGGACATCGTTCAATAAATCCTAAGTGTCTTTTTT
ATGAGGGTCCAAAATTTAAACTTATCACTAGTGTTAGAAAAGCCAAAATATTTATTTCAAGCAACTATTTCT
TTGTATCTTGTCTGCTAGGATACCAGAGTCACATCTTCTACCACCCTCTTTTTTTTTTGAGATGGAGTCTCA
CTCTGTTGCCCAGGCTGGAGTGCAGGGGCATGATCTCACCTCACTCCAACCTTGCCTCCAAGGTTCAAGTG
ATCCTCCTGCCTCAGCCTCTGAGTAGCTGGGATTACAGGTATATGCCACCATGCTCATGAAGTCTACCATC
TTATAATGACTGCATAGAAATACAAGGAATGAATACATTGACTTCACTTCTTTTTTTCCTACTTATGTTTTTT
CTTTACTCTGGTTTATGTTTATTTTTAAAAACTCTGTTATTACTTGTGTAATGATATAAATCATGTCACATTG
TTGTAATAGAGTGGAATTGAAACACATGAGCACAACCCTCTGAAGAACTCATAAACCTCTTCATCTATTTAC
TTATCTTCATGTGCTTAGTTGAATGATCAAGTATCTTAAGGAGAGAGGCCTGTTTTCCTGATGTTGGCAACT
ACACTTACAGCCAGATGGTACACATCTCAAAACATGGCCTTTCAAACACTCTGCTCATGAAGTTGAGGAGA
GGAAAGGAAAATTTATTGAGAGACAGCTTCAATCCACAGAGACAAAAGTATTGTCATCAAATGCTGCACAG
CCTTAAACCTTCGAGGCCCACGAGTCTGGCCTGGTGCCACAGCCAACCATTACCGTAGGGGAAATTCAGTG
TCTGACAAACATTTCCACTCTAGTCTTCATGCTCATGGTGTTCTTGGGCGGCCATCCAGGCCTCAAGCCAA
AATGAGGTTTCACTGTGGCCACACTCTTGATAGACCAGAGGTTCTGATAAGTATCTTCAGTTCAGATCATC
CTCCCTATGTCACTGTGGTCATGGCAAAGTAAGCACAACGGCTATGGTTAACATTTGTGAAGAAAACTTTT
TGGATCACTATTACAGGCCCTGCTGATACCTCTGGGCCCAAAGGGCTTTGTATTTCAGCATAGCTACTTGC
TTTAATGTTTCATTTCCTCAGCAGTTTCAAAACTACAAAGATGACAACAGTGGCAGATGGGACCTGACGCT
TGAACTTGGGGAGCTTTCTCCAACAGGATGAATCCATGGAAGACAGGCACATTCTCAAAAGAACAGTTTAG
CTGGTGGAAATGAGAGTAGGGTGGATGTGTTAAAATTGTTGATAAGTAAATACCCTGATTGGGTATTCCTG
TCAGGTGCTGTATAAAAAATCTCTAAAACACTGGGTGAAGTGATAAATGGATTCCATTTACCGGTGAGCAT
TTACTGGGGTCTATTAGCTGACTATTAATTAATGATTATAATATAGGCGGCAAATGGGCTGTCATCATAAAA
GGTGAAAATGATCAAGGAGAAAGAAGTATGAGCTCAGAAGAGGATGCAGCAATGTGATGAGACAGAGTTG
TGCCTGTTGGCCTCCTCTTATGAGAAGGCTTACCTGAAATTATCACTGAAGCAGGAAAAACAGTAAGGTCC
TTATTCTTTTGTTTATTCAAAAATGCAATAGCACACTGATGCATATGGAACCTGGGGCTATGAGAATTGTAA
AAATTGACCTGGGATAGTTGTGTTGGGGTTATCGAACAGAATGCTGGTATTGGGGAGGATCCTGGGGACAC
ATGAGGTGTTAGATCAATTGGGGACTGTGGACTTCTTGTTTCCAGGTGAACCTTGGAGGTGGGTGTGAGGG
GTTGGTAAAAACTTGAACTTGACCTTCCAGTGAGTCCAAATGGCTTTGACAAACATGTGGTGGGCAAGGCC
CAAGAAATGAGAGGAGAAACACTTAGGAGCCTGAATGAAGTAGGAGAGTTAGATGTGTCAAGTGTCTCTGA
AAGTAAGACGTCGAGGAGCTCAAGTAGGTGTGGTCCATGGTCTCATGTATGCCAGAGACTCAGAGCAGGG
ATGGCATCTTGAAGGAAGGAGAAAAGCCGAATGGGTCAGTAGAGACACTGGCAGACAATGAGGATGTGCA
AAGTTGCCAATACATAAGATATGGCTTGCCTGTGTCCCCAACCAAATCTCATCTTGTAGCTCCCATAATTCC
CACATGTTGGGGGAGGGACCTGGTGAGAGACGATTGAATTATTAGGGTGGGTCTTTTCCTGTGTTACTTTT
GTGATATGAATGGCTCACGAGATCTGGATGGTTTTTAAAATGGAGTTTCCCTGTACAAGCTTCTCCTCTCTT
GCCTACTGCCACAAGAAGTTTTCAATTATTATGCCTGAGACAGAGTCTCACTCTGTTACCCAGACTATAGT
GCAGTGACACAATCATAGTTCACTGCAGCCTCAACCTCCTAGGCTCAAGCGATCCTCCCACCTTAGCCTCC
CAAGTAGTTAGGACTACAGGTATAGGCCACCACCTGGCTAATTATTTTATTTTTTAGTTTTCATAGATGGGG
TTTCACTTTGTTTGTAATGTCATTGTTCAGTTCATTCTTGAATGGAATCTTACAGATTGCCTTCCCTTTTTTT
TGCTTTCCTAGAGATACTCTTATACCTGTTTTTTACCAACCAAATTTTATAATTTTATGTTATAAATAATTTT
TATCTTTTCACTTTTGAATTCATTTGTAAAGAAAAAAAGACTTGAAGGAAATGCACTATACTATGTTATTAG
TGATTATCCCTGAAGGGTTGTAATAGATGGTGGCCTCTCCAGTCTTCTGTATTGTTTAAATTGTCATCATGA
ACACTACTACTTTTATCATCAGATCTAATTGATGAATGCTTATTTTTAAATTTTGTCTAAAAGTGACTATTCC
CTAATATCTTTTTAAAAACCATTAAACTTTCAGTTTCCTTCAATGCATGTACATACCCTTGTTGAAAAAAAA
GCTATAAAAAATTAAAAGCCTTCAGAGATTTGTCATTTATTTATACATGGCATTAGTGTCCACATTATACCA
GAGATCACATACTGTGAACTTCATTCTGTACAGTGCCTAGTTGACTACCTGTATTCAAATACCAAGGAACA
AAAAGTAAAATAGCACTTCTATCTTTTTCATTTCCTGTTTCTTCATGTGACAGTTTATAAAGGTATAAGTAA
AGTTAAGAATATATGTATATTGTGCAAATACATAACATTTTTAGGGATATATTATGTGTCACCTACAAGTTC
TACAAAGTATTTTTTCCAGCAATCCTGAATCAATTCTGTATGGGAACAAAAAGGAAGGAAAAATTTAGTAA
AGTGCTTAACTTTCATATGGTGATGCCTGTAGCTCTGGCCTATTCTATAATCGGGCTGTGTACTACATGTAT

CTTAGATAGGACTATTTATTGTACCCATTGTTAGTGCAGTTGGTAGTTTCAGCAGCTATTCAAATGTCTTCA
TATTAGAAGTATCTCACATGGAGCTCAAGTGAAGGTGGTCCTTTGCCTGTGAAGAATCTTCACATTGCTAT
AACAAACTACCTGAGACTCGGGCAATGGAAAGACAGAGGTTTAATTCCACTCACAGTTCCTCAGGCTGTAC
AGAAGCATGGCTGGGGGAGGCCTCAGGAACCTACAATAGTGGAAGGCAGAGAGGAAGCAGGCATGGTGCT
GTACCACTGCTGAGCAGGAGGAAGAGAGAGTGAAGGGGAGGTGCTACACATTTTGAACAACCAGATCTTG
TGTGATCTCACTCACCATCATAAACAGCAAAGGGGAAATCCTCCCTATGGACCCAGTGACCTCCCACCAGG
CCGCTTCCTCCAACACTGGGATTCCACTCCACCTGGAGATCTGGGCAGGGACACACATCTGCAAACCATAT
CACCCGTGGAAATATGCTAGTGTTTGACAGTATGACTATAGTGTGCAGAGGGACTTTCAGTCATTAAAATC
AAGGAATTGAGTTCATACCAGAAAGTGTAATTTCTAAATTTTAATTTGCATTTTAAGAAATTAAATGCCTGA
GTAGAAAAAAGCAGTTTTCTTCAATATATTAGGAGTGATAGTATACTGGAGGTGAAGGGAAGTTAGTTAAT
ATTTAAATGACCTTATTTTACGATTTTATGAAAGGTTGAGAGTTAAGCAAATGGAAAGATTTGCCAAAATTA
GCACATGATATAAAACTGAAGAAATTTAGGAAAGTATTCAAAAATTAAAGAAAGTAAGTTTTTGAGAAAAT
TTTCAGAAAAGAATTGAAAAAAAAAAAACATTTTCAATATCCTTTATTAAAAGCTGTAGGGTTATTTAAGAAA
TCTTTTTATTTCCCAAGATCATAATGATATTCTGTTTTCTTTTCTGGAAACATTGTTTCAAGATTTTATATTT
AGGTCCCTGATTTCTCTCAAATTATTACTCTATTTTTGAGACAGAGTCTCACTCTGTTACCCAGACTATAGT
ACAGTAGCACAATCACTAGCTCACTGCAGCCTCAACCTCCTAGGCCCAGCAATCCTCCCACTTTGGCCCTC
AAGTAGCTAGGACTACAGGTACAGGCCACCACACCTGGCTAATTATTTTTATTTTTAATTGTTTTTCAGATG
GGGTCTTATTGTGTTACCCAGGCTGGTCTTGAGTTCCTGACCTCAAGTGGTCTTCCCACCTTGGCCTCCCG
AAGTGCTGAGATTACAGGGTGAGCCACTCCCAGCCTTAAATTAATTTTTTGTATGGTGTGTAGGGGGATTG
CCATTTTATTTTACATTGAATTAATGGATTTCTACTTATTATTGAAAAGATCATCTCTTCTGTAATGAATTGC
AGTGAGTCTGTTTCATAAATCATGTGATGTATAAAAATTTGAGACTGTTTTTGACCCGCTTTGTCTATTACA
TTGGTCTATTTATCCAGTTTCTCGCCCCAGCATAGCACTGTATTGTTACAAGGGCTATACATCTCCAGCCTT
ATTCTTCATTAGCATTATTGGCTATCCTAGTTCCTTCCAATTAATATATAAATTTTAAAATCAGCTTGCCAAT
TTCTATAAAATAAATATTGTGAATTGATTAGATTGCATTGAATCTTGGGTTCAAATATCCAAAGATTTCATT
ATTGCTGTTACTTGCTTTGCACTACAATTATTTATTTGTTGTATAGTTTGTATGTCATGTGTCTGATATAACT
GATCACATATGTGGGTGCATTAATTCGGCTATCCACTTGACATGTTTAAATTTTTTTACTGGCCGTATTGAG
GTAGACTTGACAAAAATTCATATATATTTAAGGTGTGTAACATATAAGTGTTTTGATATAAATGCATACATT
GTGAAATTAATACCATAATTAAGCGAATTATGTATTTGTATATATCTATATCTAGATAACCTTATATAGTTA
CAACTTTCGATTGTGTGGTAAGAACACTTAGATCAACTCTAGCAAATTTCAAGTACAGTACAGTATTATTAA
CTATAGTCACCATGCCAAGTGTTAGGATCTCCAGAATTTATTCTTCCCACCTAACTGAATCTTTGTATTCTT
TAAACAACATTGATTTATTCAACTCATGAATGTGGTATGTCTTTTCTCTTATTTTATAATCATTTCTTTCTCT
TTAGCTGTGTTTTTGAGGATTTTTTAGTGTTGATGTCATGTAGACATTTTTATTCAAACATTTAATTCTTGAT
GCGATTGTGCTCTAGTATTTAAAATTTACTTTCCACATGTTTATGGTTACTATATAGAAATATACATTTTTAG
AGTAATTTCATCTCTTATTCAACAACCTTCTATTCATATCATTTTTATTAAATTGCTCATAGATCTTTTAAAT
TTTCACATGTATAAGTTTGCTGGGGTGGTGGGGTTCTCATGGAGAGCTTCCTCTGTTAGGGTAGGGTGGAA
GGGAAATGTTCGAGCCCCCATCCAGAGTCCACCTCAGCACTGCCTAGTGGAGCTGTGAGAAGGCTACCAT
CCTCCAGGTCTTGGAATGGTAGATCCACCAATAGCTTGCATCATGCCTCTGGAAAAGCTGCACACTCTCAA
CACCCAGCTTGTGAGCAGCCAGAGTGGGCTATACCCTGCAAAGCCACAGGGGCAGAGCTTCGGGCTGTGG
GAACCCACCTCTGTGATCAGTGACCTGCATGTTAGACATGGAGTCAGCGAGATCATTTCTTTTTCTGAGCT
TATGGATTTGATTGTTCTTGCTGGATTTTGGACTTGCACAGGACTTGTAGCCCCTTTGTTTTGGGCAATTTC
TCCCATTTGAATGGCTGTATTTACCCAGTGCTTGTACTCCCATTGTATTTAGGAAGTAGTTAACTTTTTATG
GCCTATAGGCAGAAGGGATGTTCTGTATTTGAGACTTAATCAGGACTTTGAGTTAATGCTGAAAATGAGCA
AGACCTGGGGACTGCTAAAGGCATGAATGTGGTTGGAAATGAGACAAGATATTTGGGAGGGGCCAGGTGA
GTTCAGGATATGGTTTGCTGTGTCCCCACCCAAATCTCATCTTGAATTTTATGTGTTGTGGAAGGACCTGGT
GGGAGATAATTTGAATCATGGGGCAGATCTTCCTGGTGTGCTGTTCTCATGAGAGTGAGTAAGTCTCATAA
CATCTGATGGCTTTAAGAATGGGAGCTTGGCCTGCATGTTTCCTCTTTGCCAGCAATACGTAACTGAGAAG
CATACCT
